# Supplementary material for: Chromosome organization shapes replisome dynamics in Caulobacter crescentus
Source: Nat Commun. 2024 Apr 24;15:3460. doi: 10.1038/s41467-024-47849-6 (PMC11043382; doi:10.1038/s41467-024-47849-6)
Supplement: Supplementary file 1 — Supplementary Information [file 41467_2024_47849_MOESM1_ESM.docx]

**Chromosome organization shapes replisome dynamics in *Caulobacter crescentus***

Chen Zhang^1^, Asha Mary Joseph^2^, Laurent Casini^3^, Justine Collier^3^, Anjana Badrinarayanan^2^, Suliana Manley^1,^*

**Affiliations:**

^1^ Laboratory of Experimental Biophysics, Swiss Federal Institute of Technology Lausanne (EPFL), Lausanne, Switzerland

^2^ National Centre for Biological Sciences, Tata Institute of Fundamental Research, Bangalore, India

^3^ Department of Fundamental Microbiology, Faculty of Biology and Medicine, University of Lausanne, Lausanne, Switzerland

* Corresponding author: suliana.manley@epfl.ch

**Content:**

**Supplementary Methods**

**Supplementary Table and Figures**

**Supplementary References**

**Supplementary Methods**

**Plasmid and strain construction**

The integrative plasmid *pDnaN-sfGFP* was first constructed by replacing the *mCherry* into *sfGFP* in the plasmid of pDnaN-RFP^1^, and was introduced into *C. crescentus* cells by conjugation with the *E. coli* strain S17-1. Notably, we tried the electroporation protocol but failed.

The integrative plasmid *P_xyl_-zapT-mScarlet-I* was constructed based on the previously published *P_xyl_-ftsZ-sfGFP* plasmid (kanamycin resistant)^2^, where *mScarlet-I* was tagged to the C-terminal of full-length *zapT* gene and then replaced the *ftsZ-sfGFP*. The *P_xyl_-zapT-mScarlet-I* was then introduced into *C. crescentus* cells by electroporation. Similarly, the *P_xyl_-SSB-mScarlet-I* plasmid was constructed by replacing *zapT* to *SSB* based on *P_xyl_-zapT-mScarlet-I*. The kanamycin resistant gene of *P_xyl_-SSB-mScarlet-I* was also replaced to a gentamycin resistant gene. The *P_xyl_-zapT-mScarlet-I* (gentamycin resistant) was then transformed into *CB15N::dnaB-YFP* cells which are kanamycin resistant. The integrative plasmid *P_xyl_-rsaA* was constructed by amplifying *rsaA* gene (from promoter to coding sequence) using the same primers as reported previously^3^, and then replacing *ftsZ-sfGFP* gene in the *P_xyl_-ftsZ-sfGFP* plasmid. The *P_xyl_-rsaA* was introduced into *C. crescentus* cells by electroporation.

The integrative plasmid harboring orthogonal ParB/*parS* system was constructed by tagging the cassette gene of “*mcherry-parB^P1^-parS^P1^*” or “*yGFP-parB^pMT1^-parS^pMT1^*” to ~1000 bp of chromosome homologous sequence from the left or right arm respectively, followed by the insertion into empty integrative vector pMCS-1 or pMCS-2^4^. Notably, the site-specific ~1000 bp of chromosome sequence was carefully chosen to make sure that the integrated cassette constructs did not affecting the expression of essential genes in *C. crescentus*^5^. The ultimate plasmids for genomic integration on left or right arm were transformed into *C. crescentus* by electroporation. To obtains strains with cassette genes on both arms (e.g. CB15N*::L1/R1*), one integrated strain (e.g. CB15N*::L1*) was transferred to another (e.g. CB15N*::R1*) by phage Cr30-mediated transduction.

To construct the *parAK20R* mutant, the cassette encoding such ParA point-mutant was transduced into wide-type *C. crescentus* at the *P_xyl_* region by Cr30-mediated transduction. To induce the ParA mutant, 0.2% wt/vol xylose was added in cell cultures 2 h before imaging.

**Supplementary Table and Figures**

**Supplementary Table 1:** Doubling time of different background strains measured in nutrient and minimal medium by microplate reader. All strains harbor the same *dnaN-sfGFP* integration.

|  | WT | *∆smc* | *flip1-5* | *rsaA* | *rsaA+* |
| --- | --- | --- | --- | --- | --- |
| M2G (28 ^o^C) | 3.61 ± 0.14 | 3.49 ± 0.10 | 3.50 ± 0.06 | 3.61 ± 0.02 | 3.34 ± 0.06 |
| M2G (32 ^o^C) | 2.96 ± 0.03 | 2.97 ± 0.05 | 2.93 ± 0.19 | 3.08 ± 0.10 | 2.79 ± 0.03 |
| PYE (32 ^o^C) | 2.29 ± 0.11 | 2.26 ± 0.08 | 2.92 ± 0.05 | 2.33 ± 0.10 | 2.14 ± 0.09 |


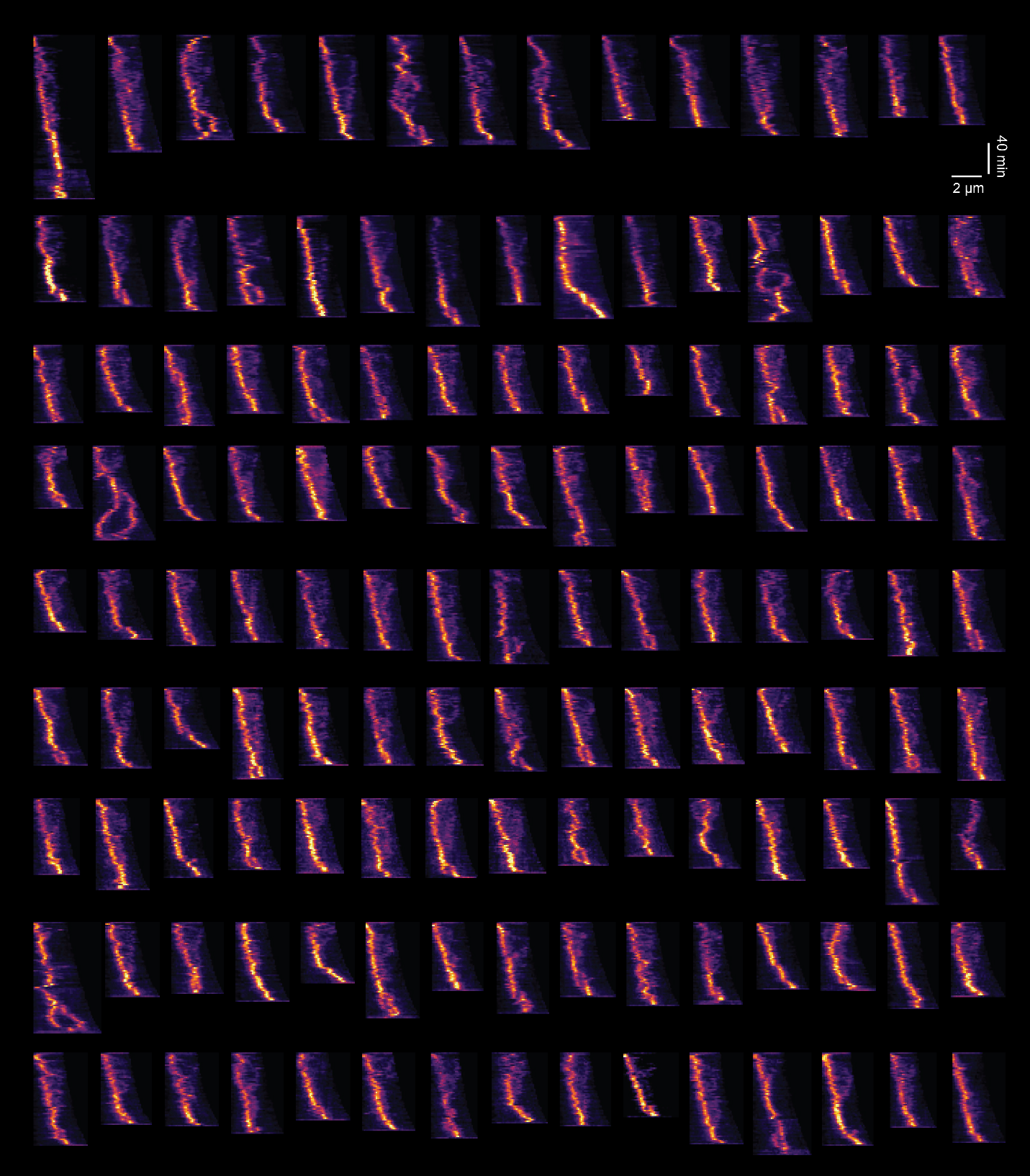


# **Supplementary Figure 1** | Kymographs of time-lapse imaging of the CB15N*::dnaN-sfGFP* cells.


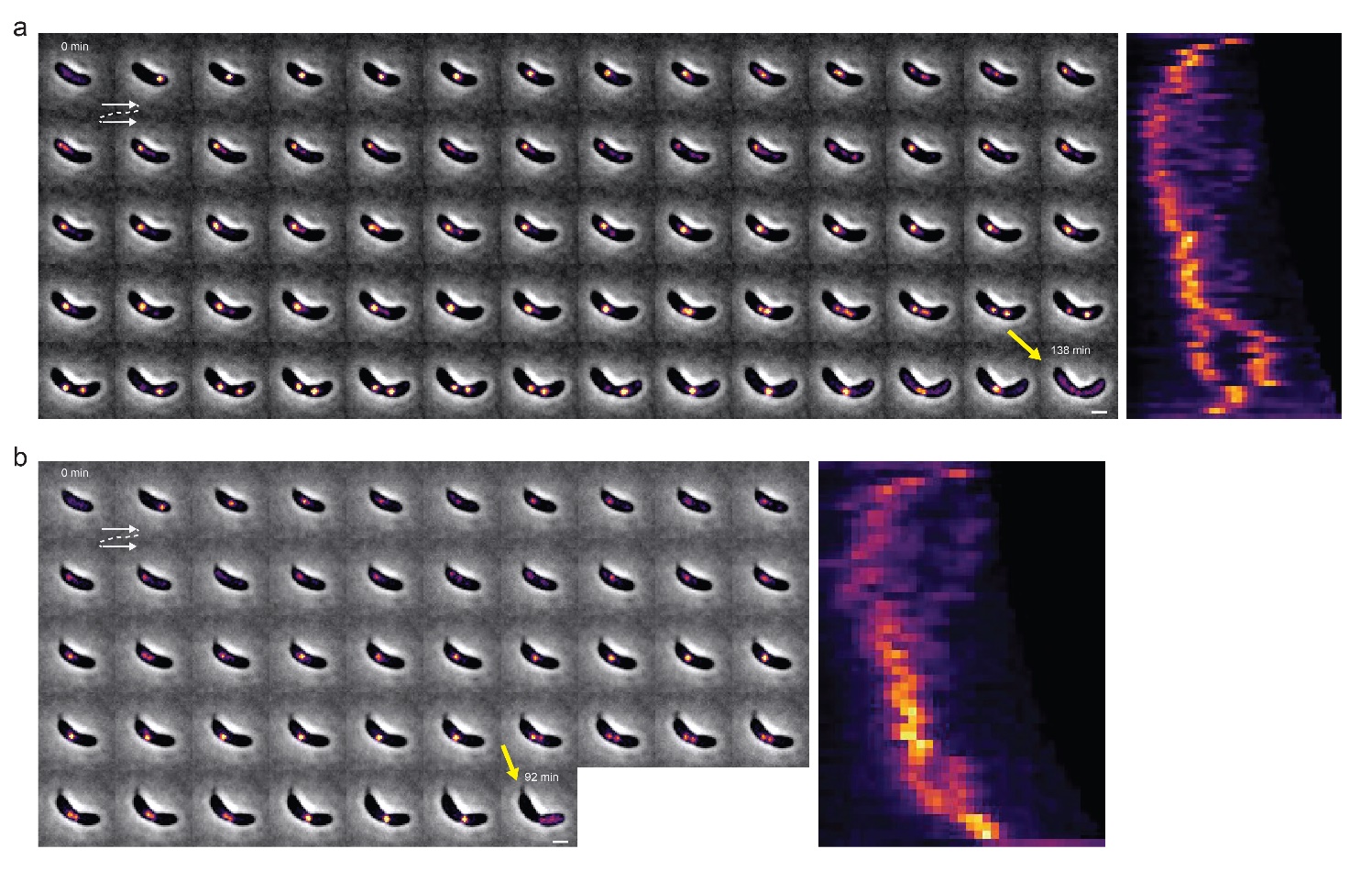


# **Supplementary Figure 2** | Montages (left) and kymographs (right) of the time-lapse imaging of two representative CB15N*::dnaN-sfGFP* cells that showed mispositioning of DnaN focus at the beginning of replication. Stalked poles are indicated by arrows.


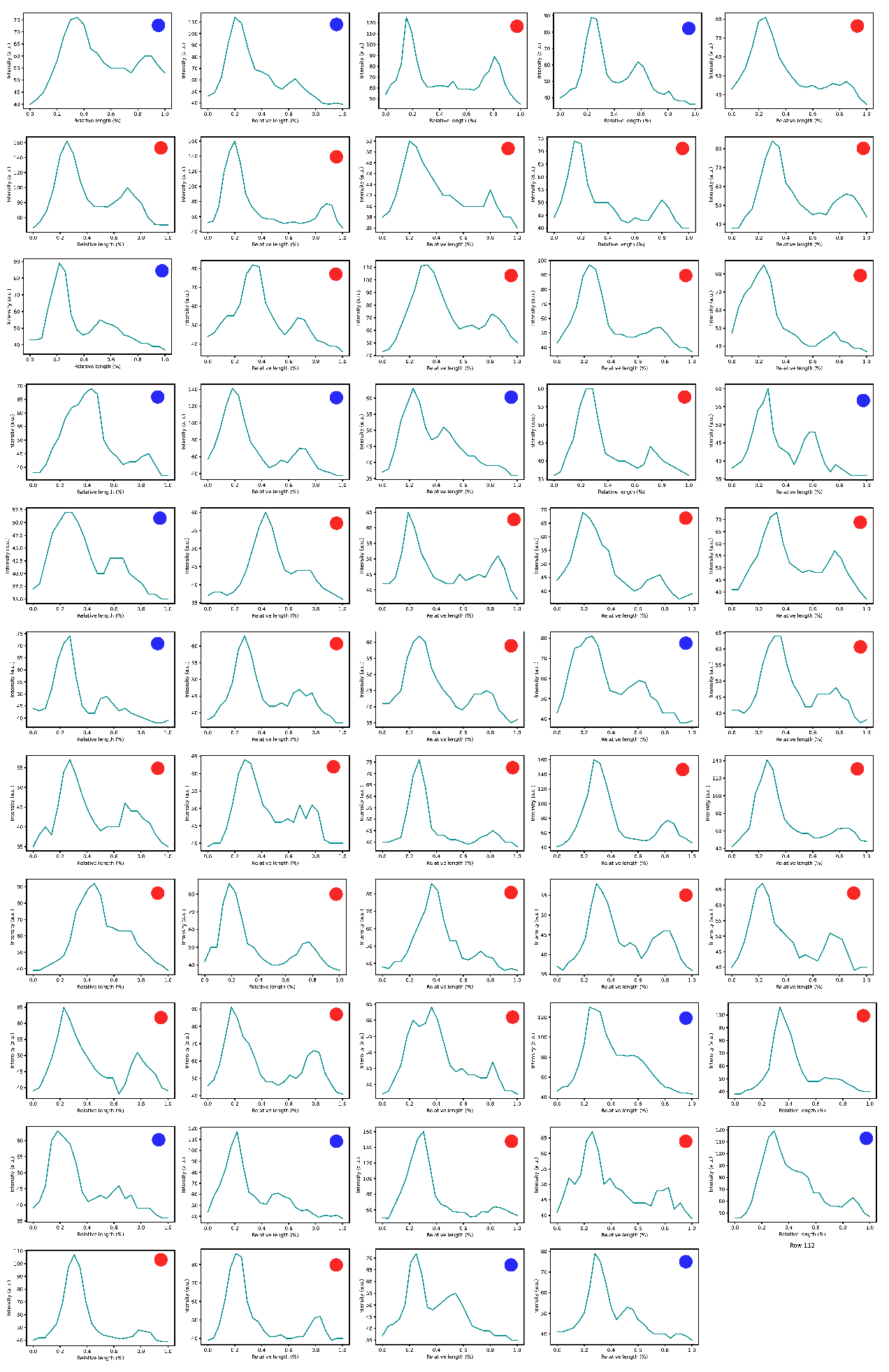


# **Supplementary Figure 3** | Intensity profiles that show bright and dim DnaN foci at 30% replication time (decomposed from **Fig. 1d** left**)**. Source data are provided as a Source Data file.


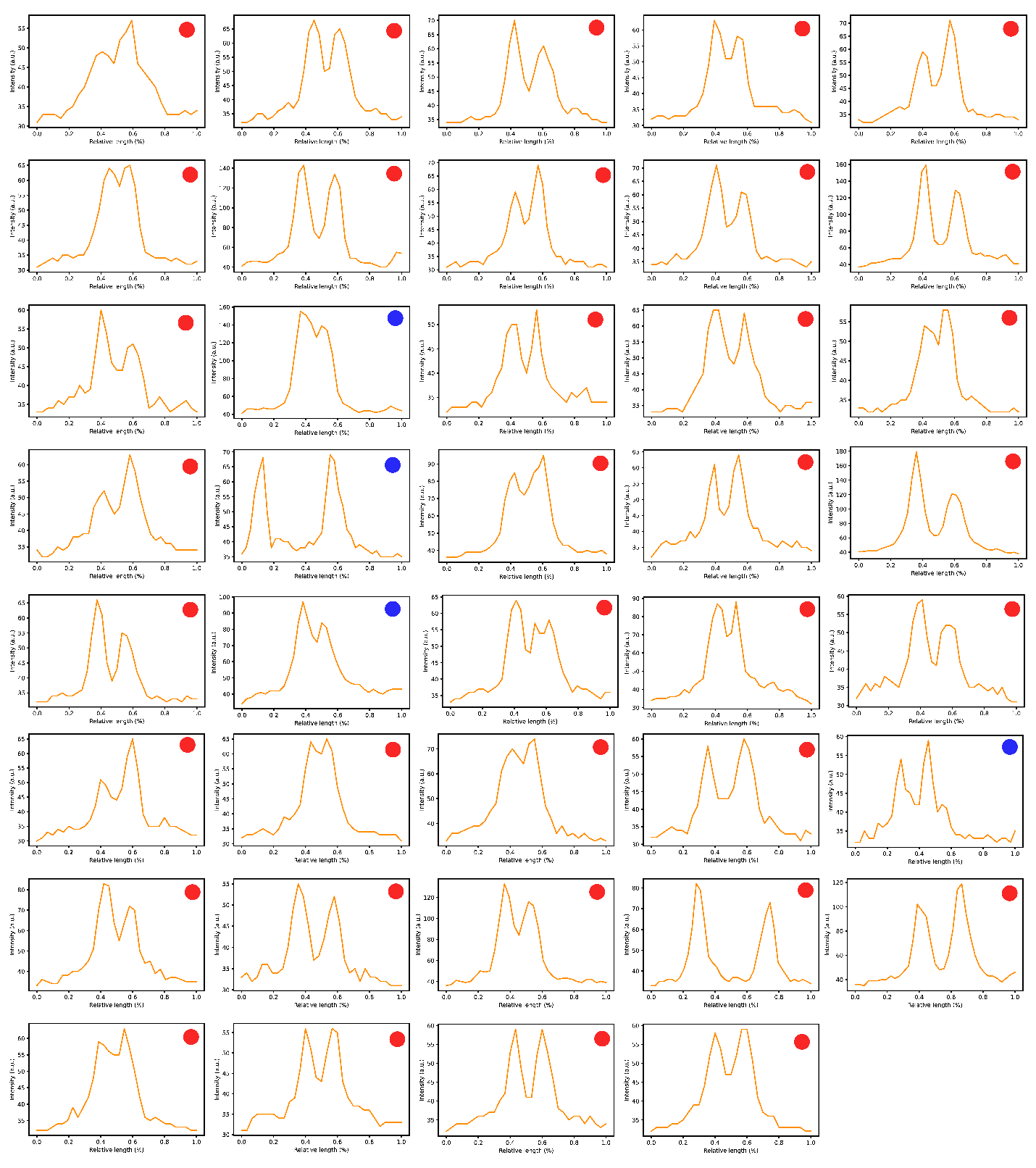


# **Supplementary Figure 4** | Intensity profiles that show two bright DnaN foci at 90% replication time (decomposed from **Fig. 1d** right**)**. Source data are provided as a Source Data file.


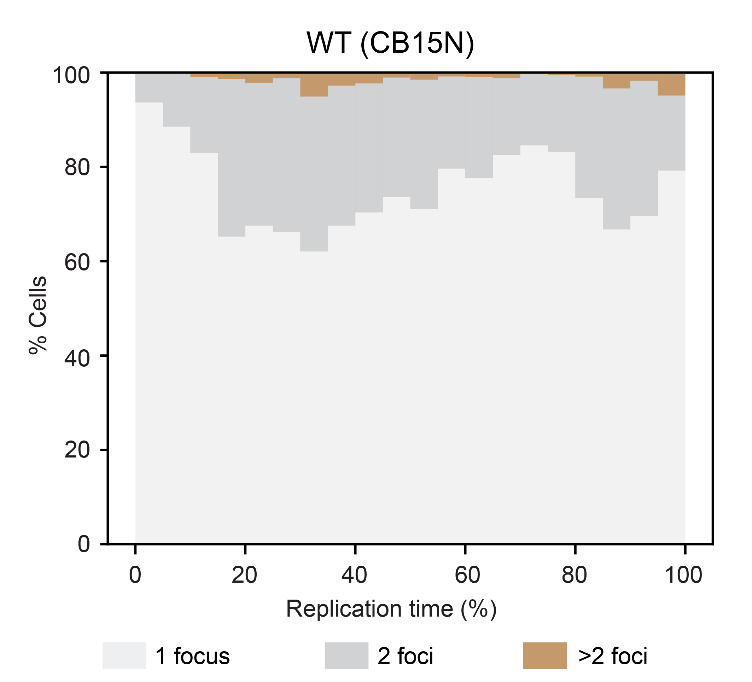


# **Supplementary Figure 5** | Distribution of CB15N*::dnaN-sfGFP* cells that contain 1, 2, and >2 detected DnaN foci. Source data are provided as a Source Data file.


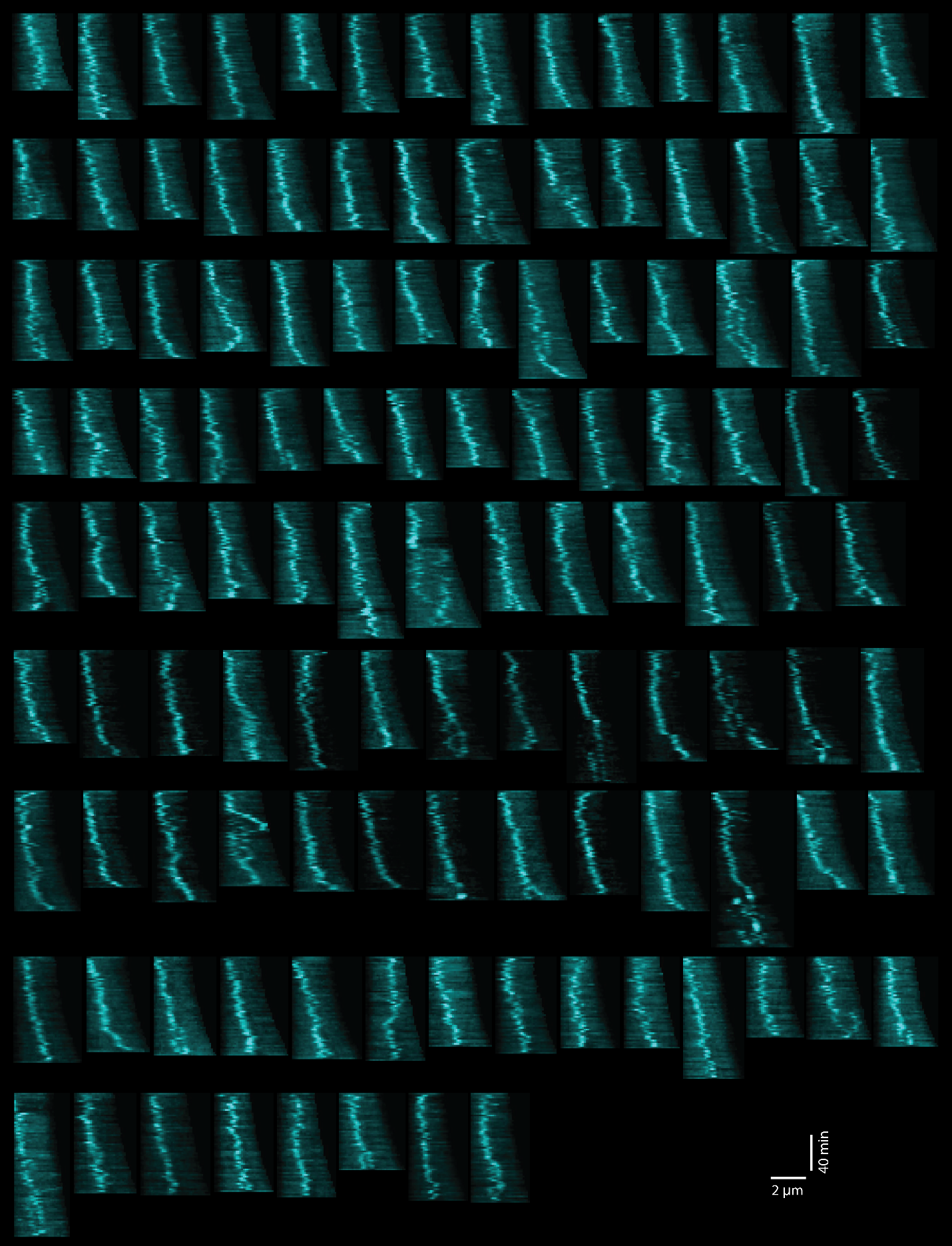


# **Supplementary Figure 6** | Kymographs of time-lapse imaging of the CB15N*::P_xyl_-SSB-sfGFP* cells.


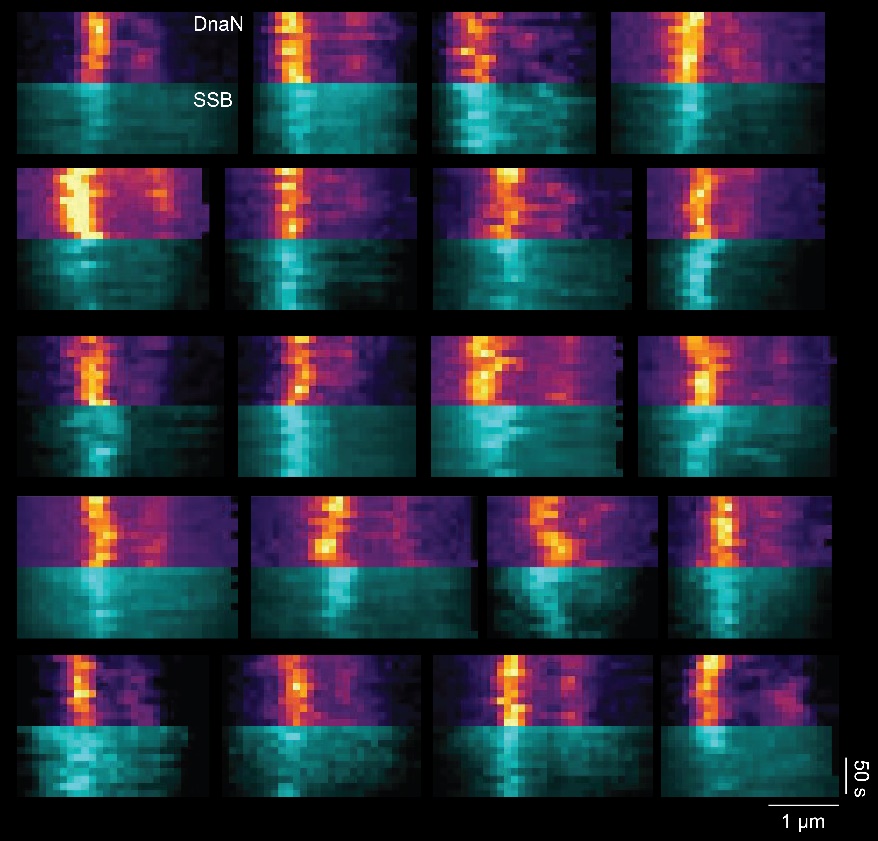


# **Supplementary Figure 7** | Kymographs of DnaN (pseudo-colored as inferno) and SSB (pseudo-colored as cyan) by imaging CB15N*::dnaN-sfGFP::P_xyl_-SSB-mScarlet-I* for 10 frames with 10 sec intervals.


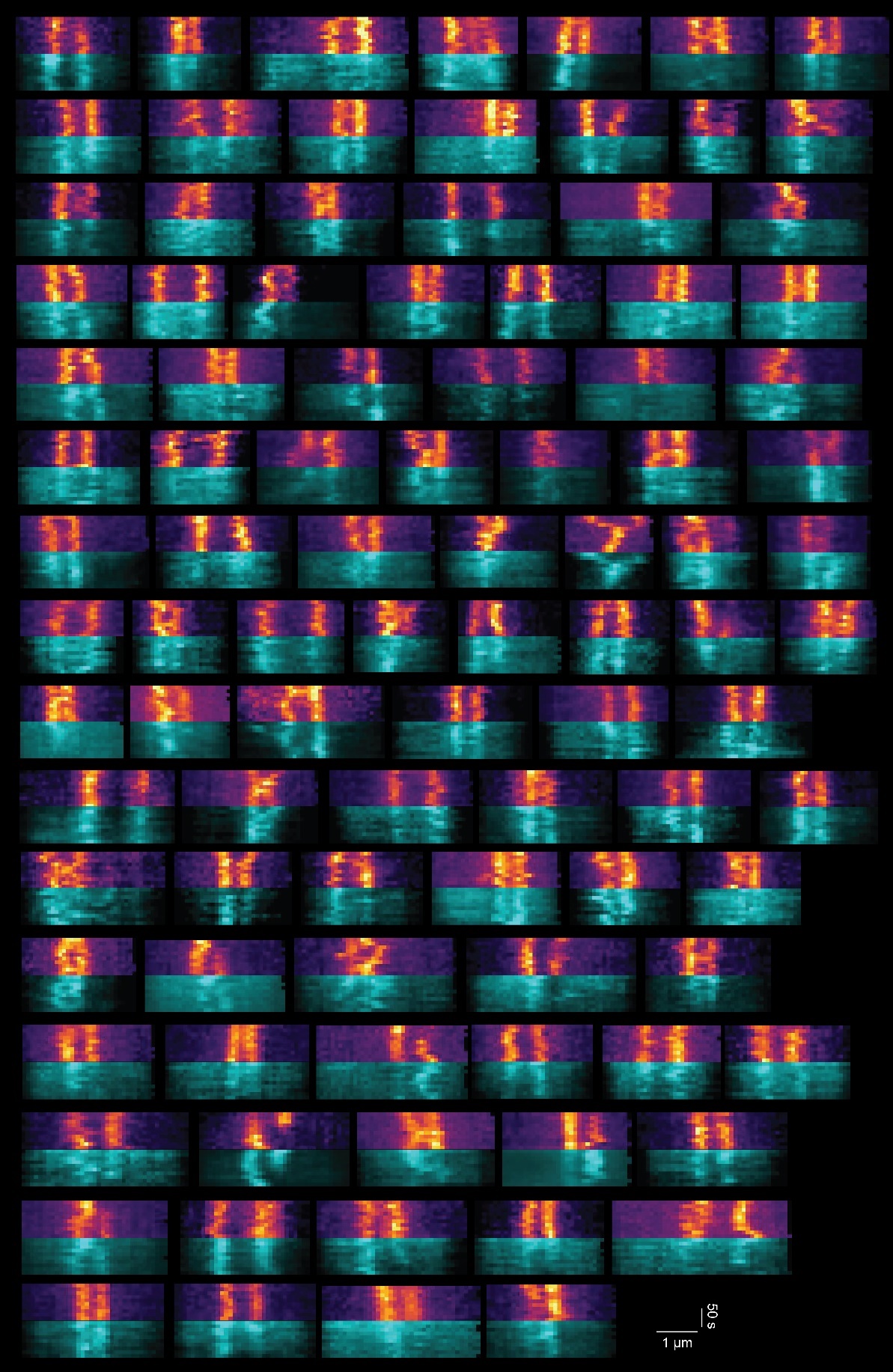


# **Supplementary Figure 8** | Kymographs of DnaN (pseudo-colored as inferno) and SSB (pseudo-colored as cyan) by imaging CB15N*::dnaN-sfGFP::P_xyl_-SSB-mScarlet-I* for 10 frames with 10 sec intervals.


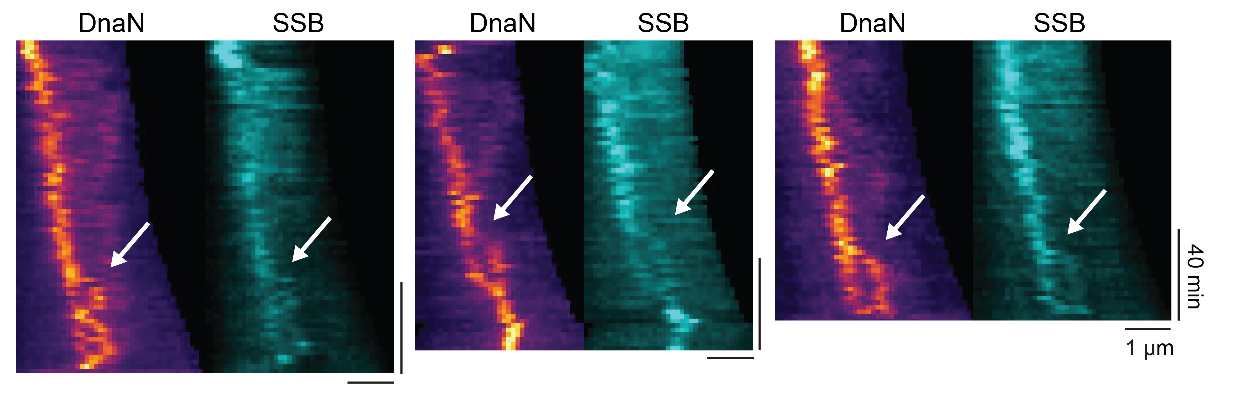


# **Supplementary Figure 9** | Kymographs of DnaN (pseudo-colored as inferno) and SSB (pseudo-colored as cyan) by imaging CB15N*::dnaN-sfGFP::P_xyl_-SSB-mScarlet-I* with 2 min intervals. late-splitting events are indicated by arrows. Identical scale bar for all three examples.


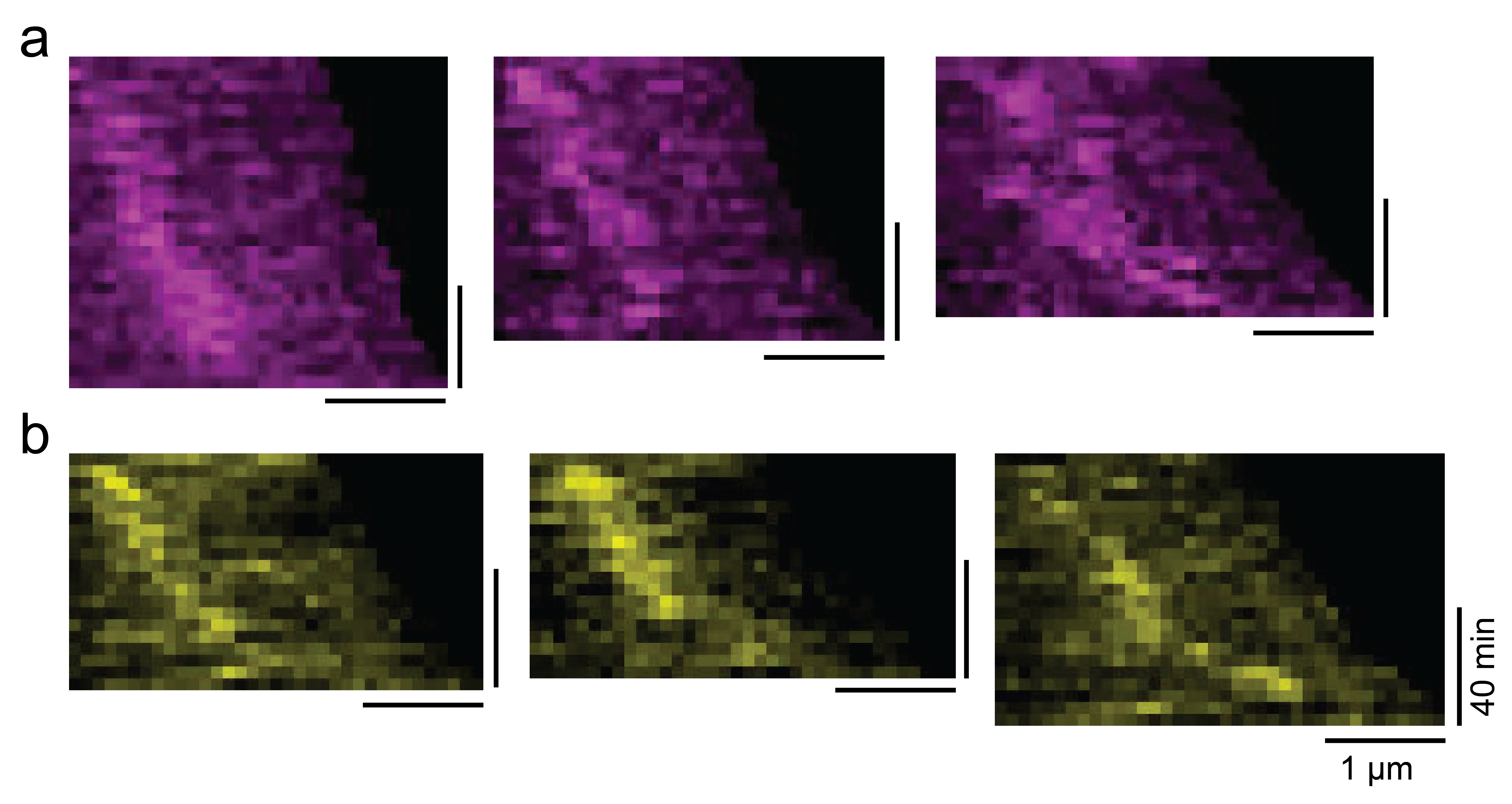


# **Supplementary Figure 10** | Kymographs of HolB-YFP (**a**, pseudo-colored as magenta) and DnaB-YFP (**b**) by imaging CB15N*::holB-YFP or CB15N::DnaB-YFP* with 4 min intervals, respectively. Identical scale bar for all examples.


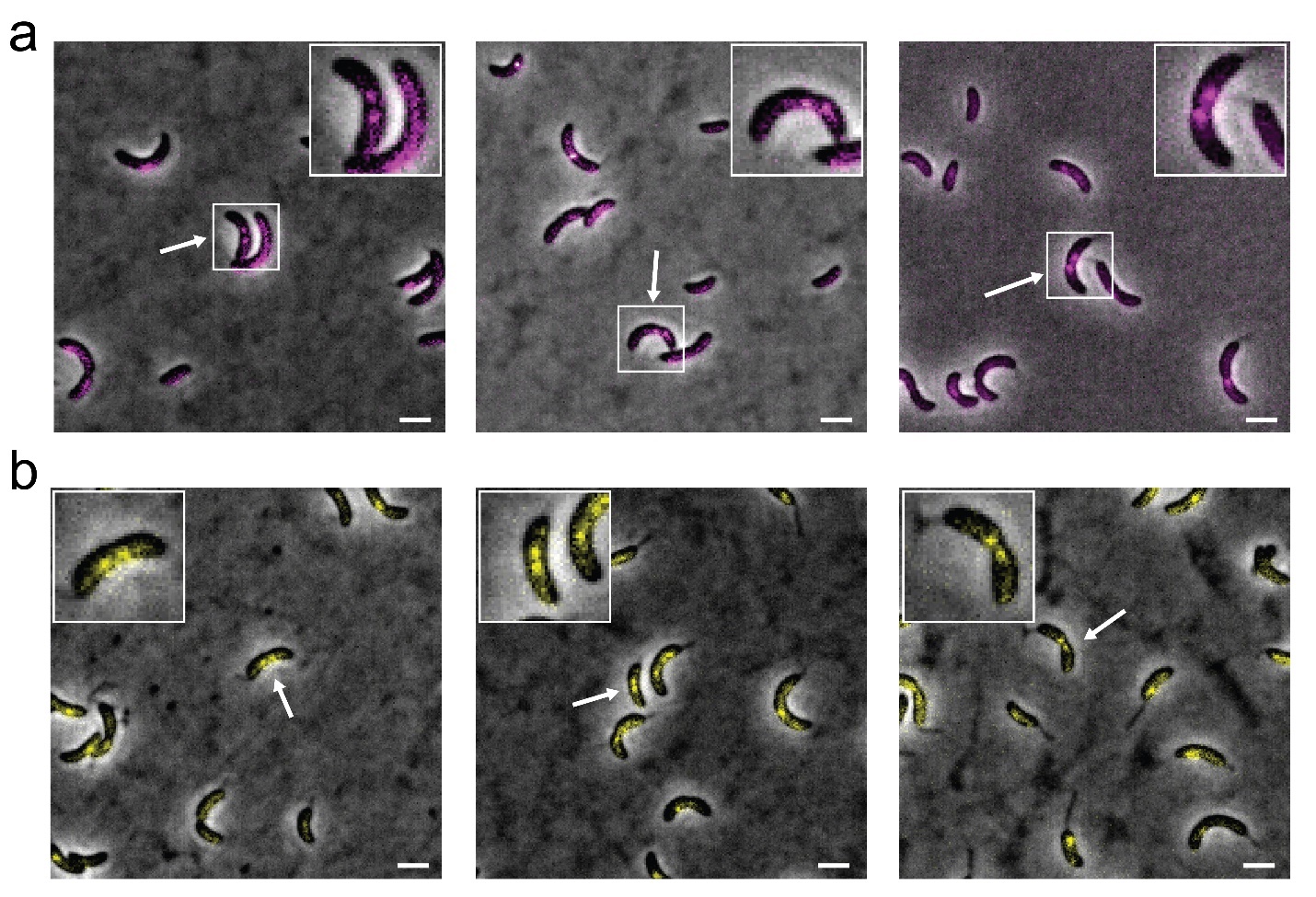


# **Supplementary Figure 11** | Snapshots of HolB-YFP (**a**, pseudo-colored as magenta) and DnaB-YFP (**b**) by imaging CB15N*::holB-YFP or CB15N::DnaB-YFP* respectively. Scale bar: 2 µm.


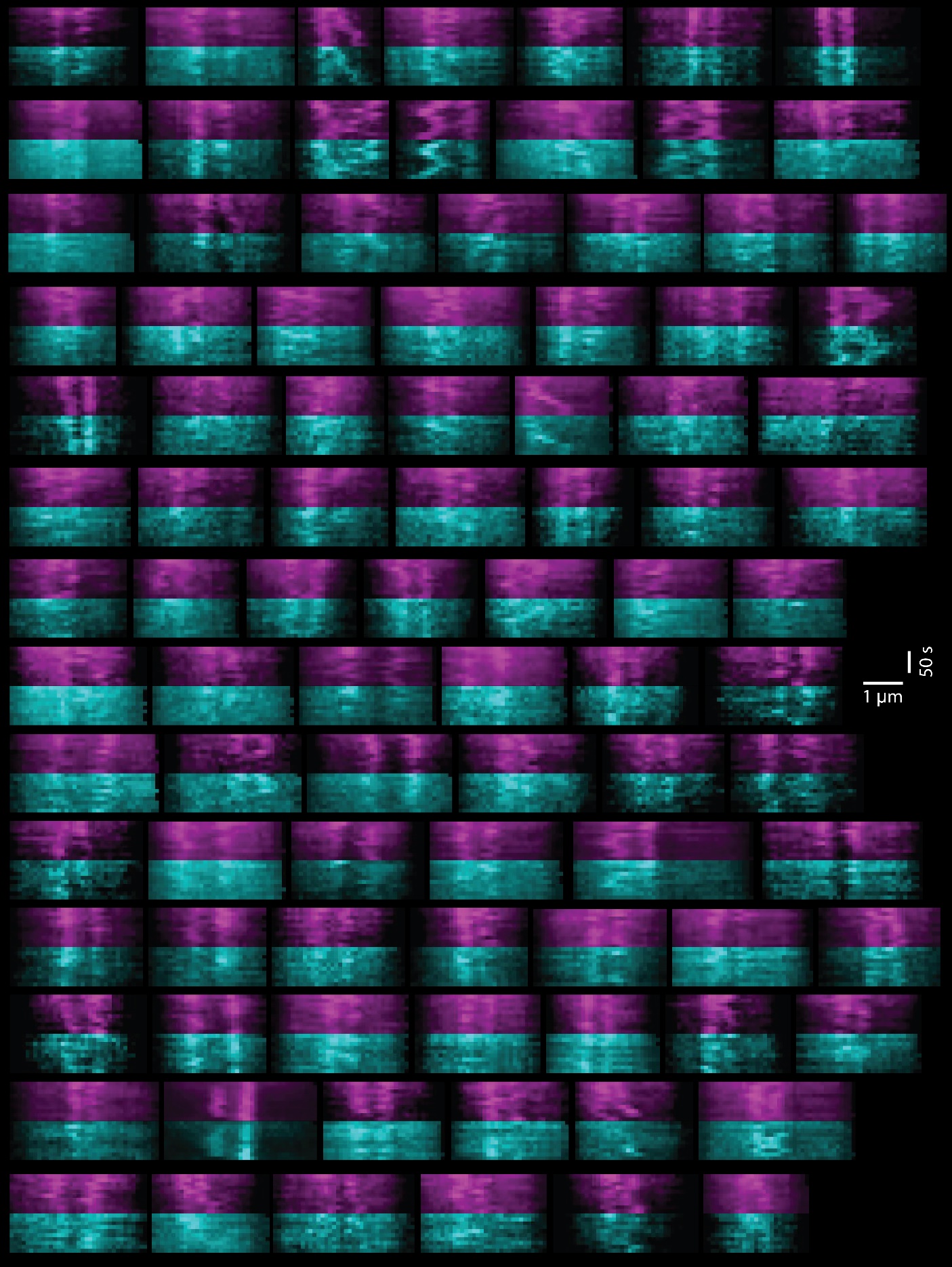


# **Supplementary Figure 12** | Kymographs of HolB (pseudo-colored as magenta) and SSB (pseudo-colored as cyan) by imaging CB15N*::holB-YFP::P_xyl_-SSB-mScarlet-I* for 10 frames with 15 sec intervals.


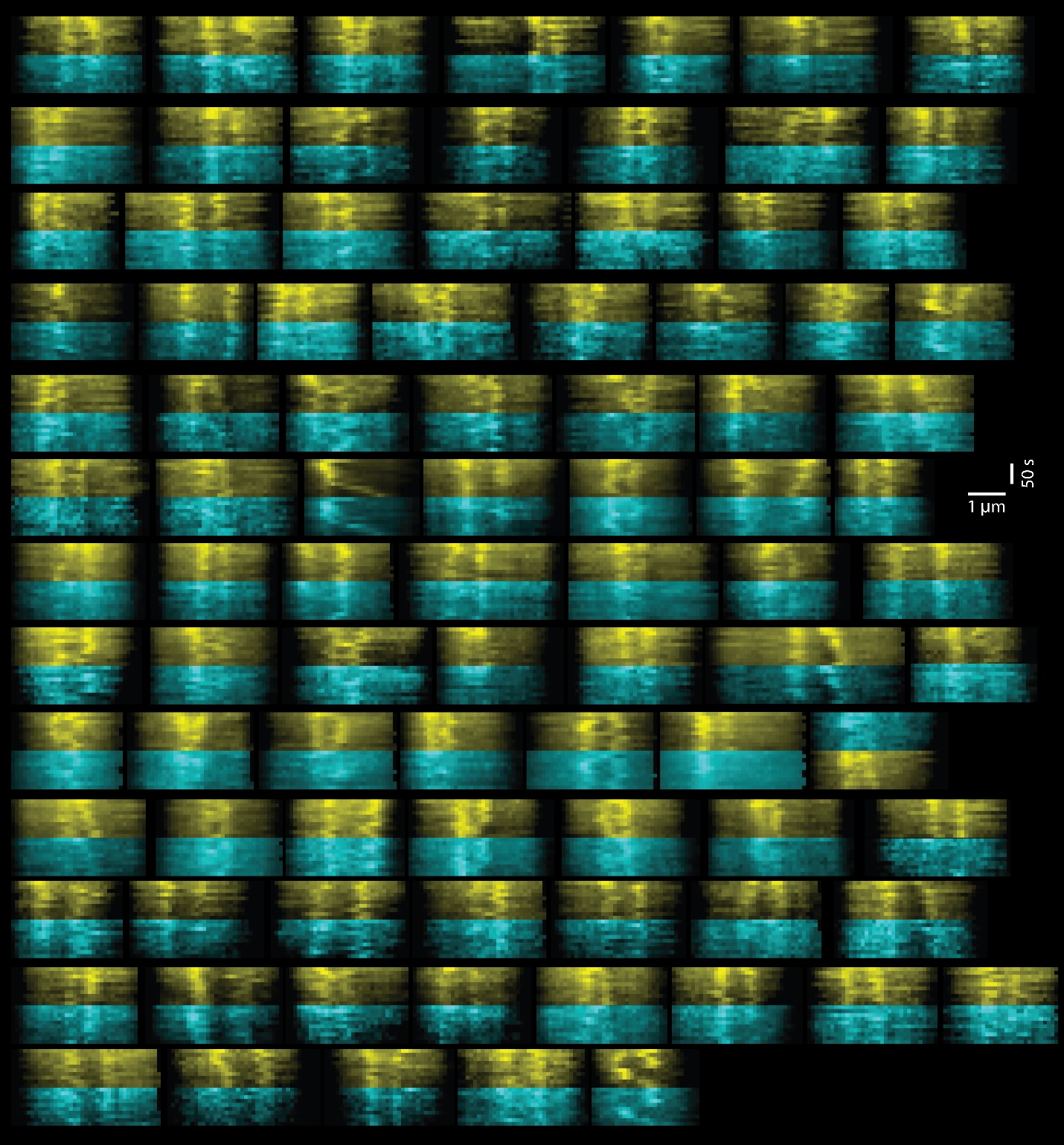


# **Supplementary Figure 13** | Kymographs of DnaB and SSB (pseudo-colored as cyan) by imaging CB15N*::dnaB-YFP::P_xyl_-SSB-mScarlet-I* for 10 frames with 15 sec intervals.


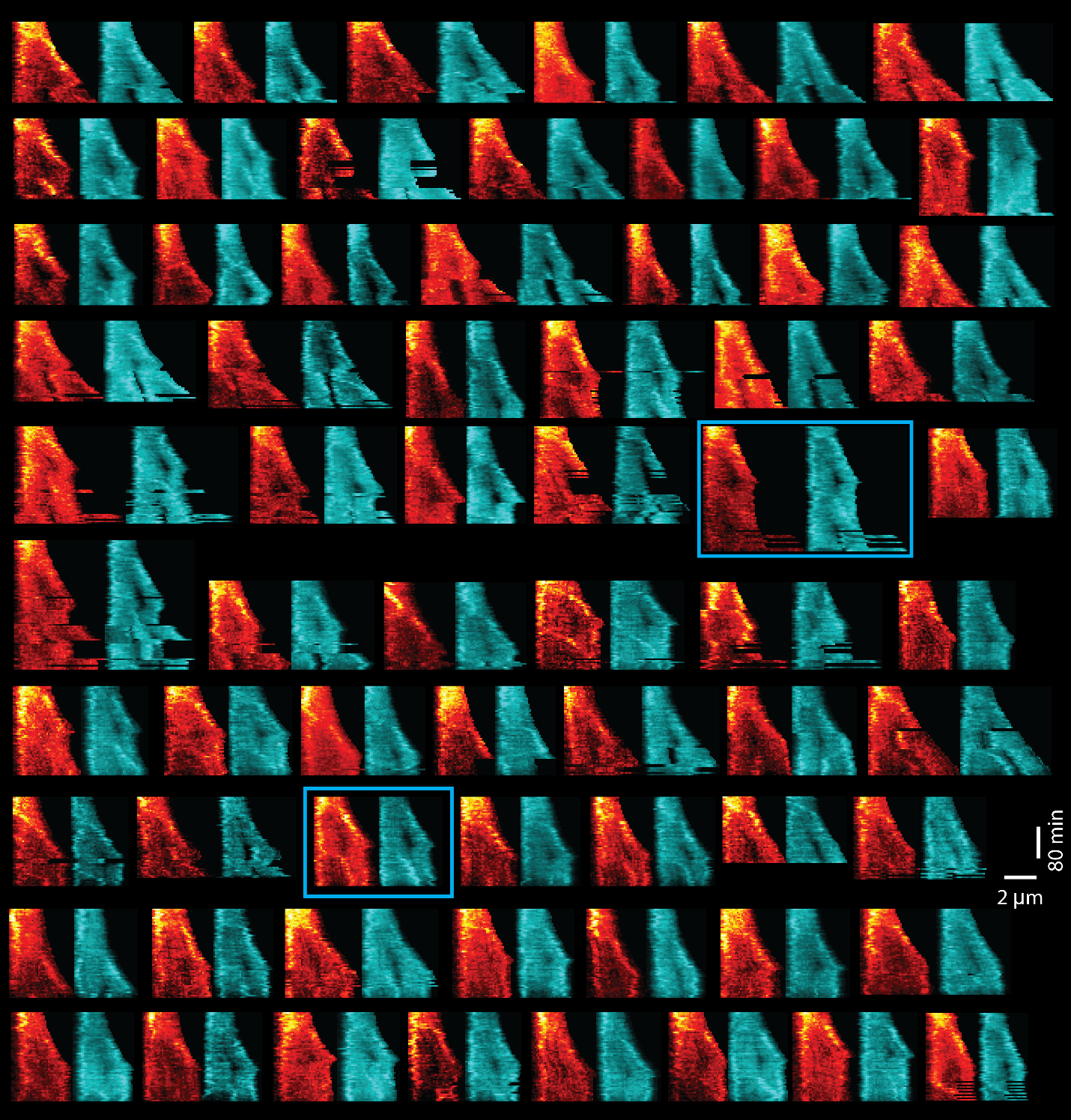


# **Supplementary Figure 14** | Kymographs of L1 (left, pseudo-colored in Red-Hot) and R1 (right, pseudo-colored in cyan) fluorescence. Two cells highlighted in blue boxes are considered to have different trajectories before loci duplication.


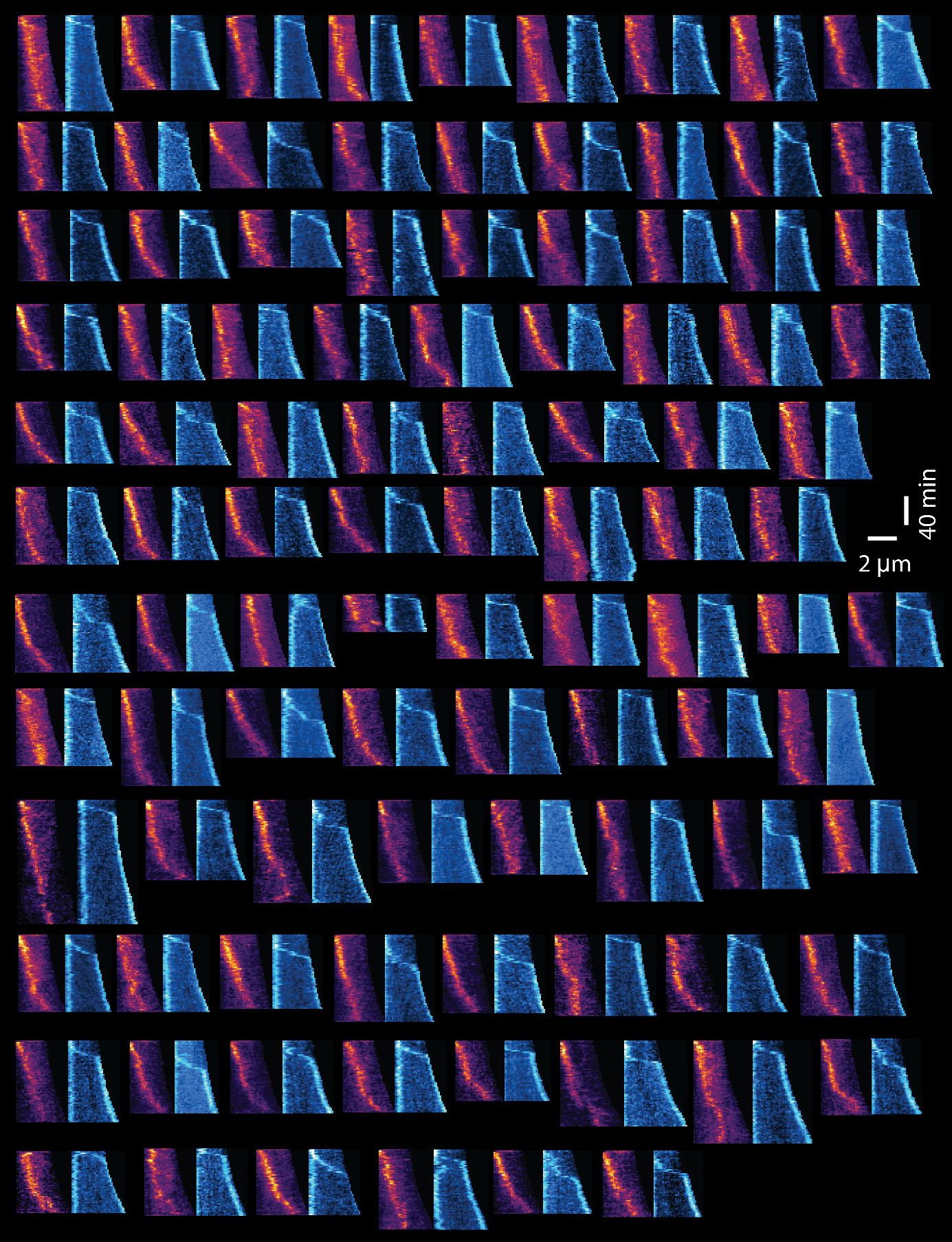


# **Supplementary Figure 15 |** Kymographs of DnaN (left, pseudo-colored in MPI-inferno) and ParB (right, pseudo-colored in Cyan-Hot) and fluorescence in WT (CB15N) background cells.


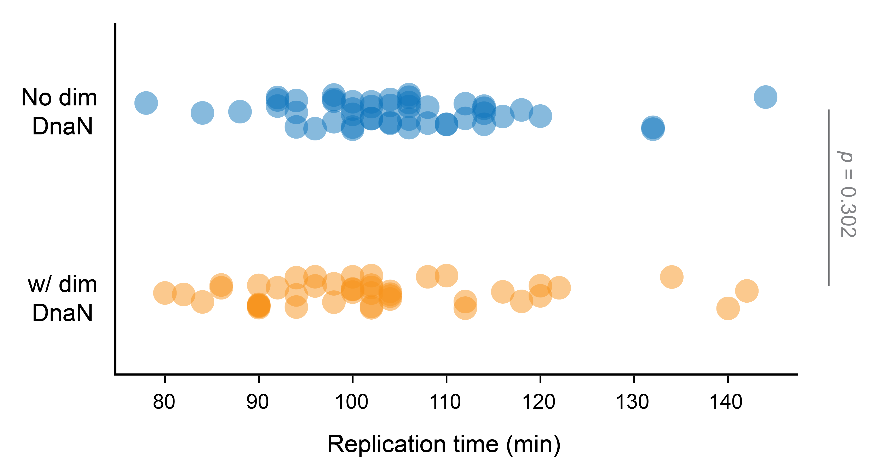


# **Supplementary Figure 16 |** Distribution of the replication time for cells with or without dim DnaN signals. The *p*-value is calculated by a two-tailed t-test. Source data are provided as a Source Data file.


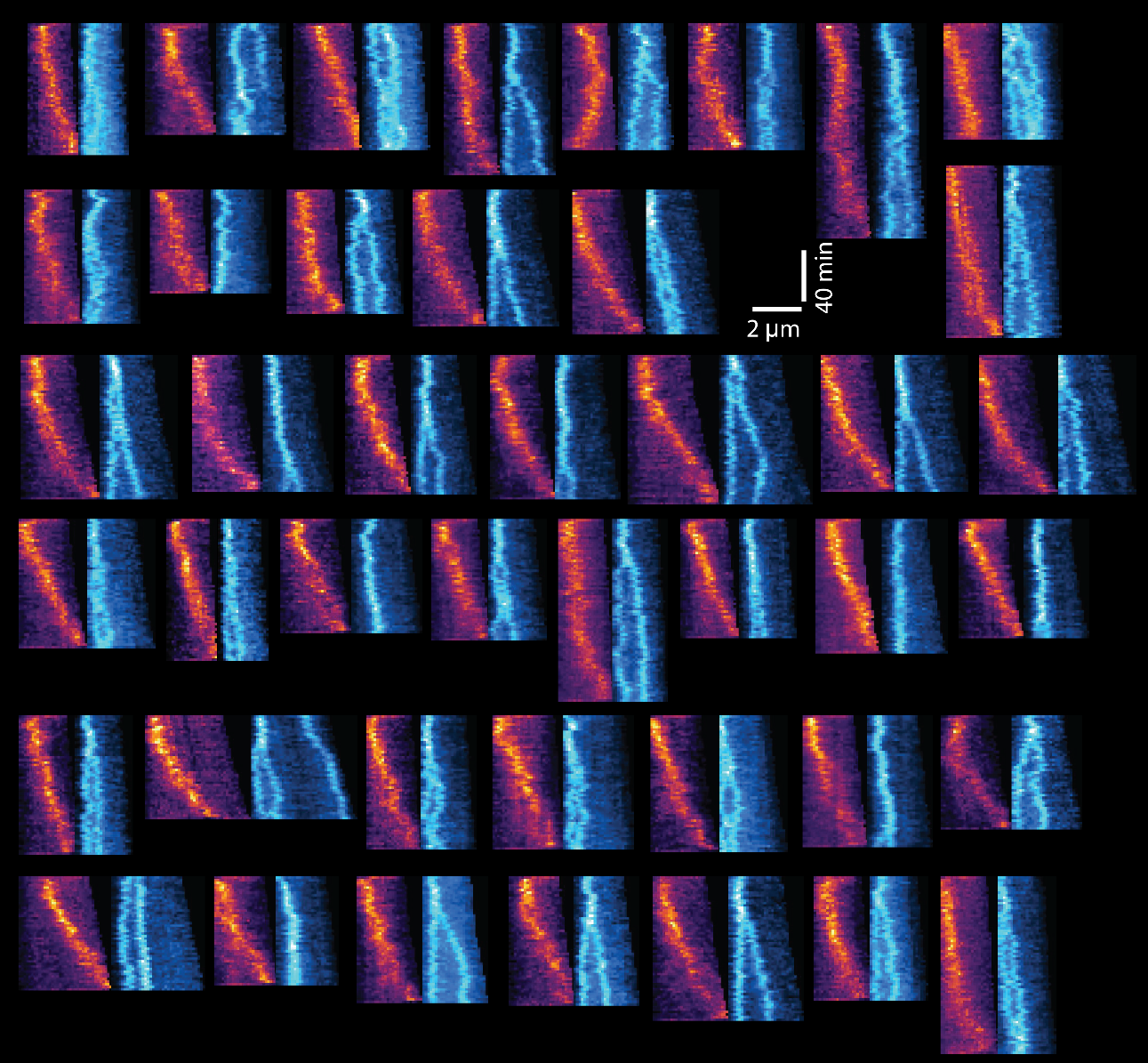


# **Supplementary Figure 17 |** Kymographs of DnaN (left, pseudo-colored in MPI-inferno) and ParB (right, pseudo-colored in Cyan-Hot) fluorescence in parAK20R background cells.


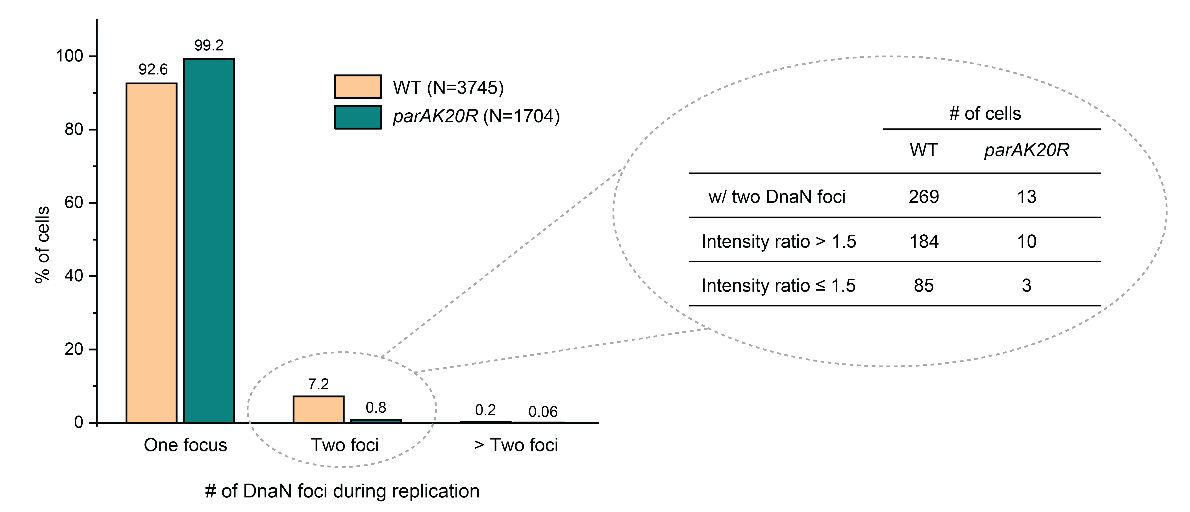


# **Supplementary Figure 18** | Quantification of DnaN foci number in WT (N=3745) and *parAK20R* (N=1704) background cells. Source data are provided as a Source Data file.


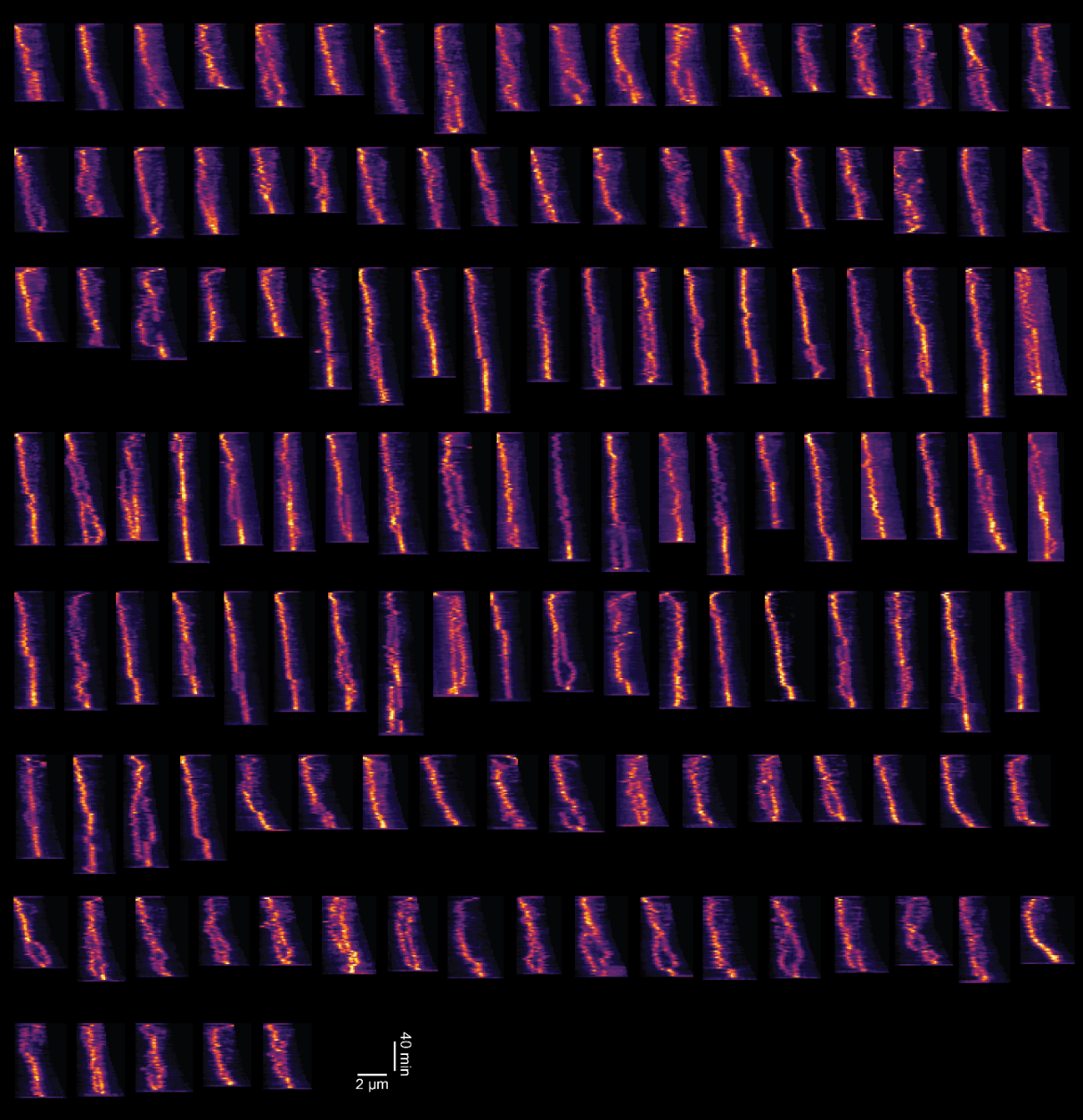


# **Supplementary Figure 19 |** Kymographs of time-lapse imaging of the CB15N *∆smc::dnaN-sfGFP* cells.


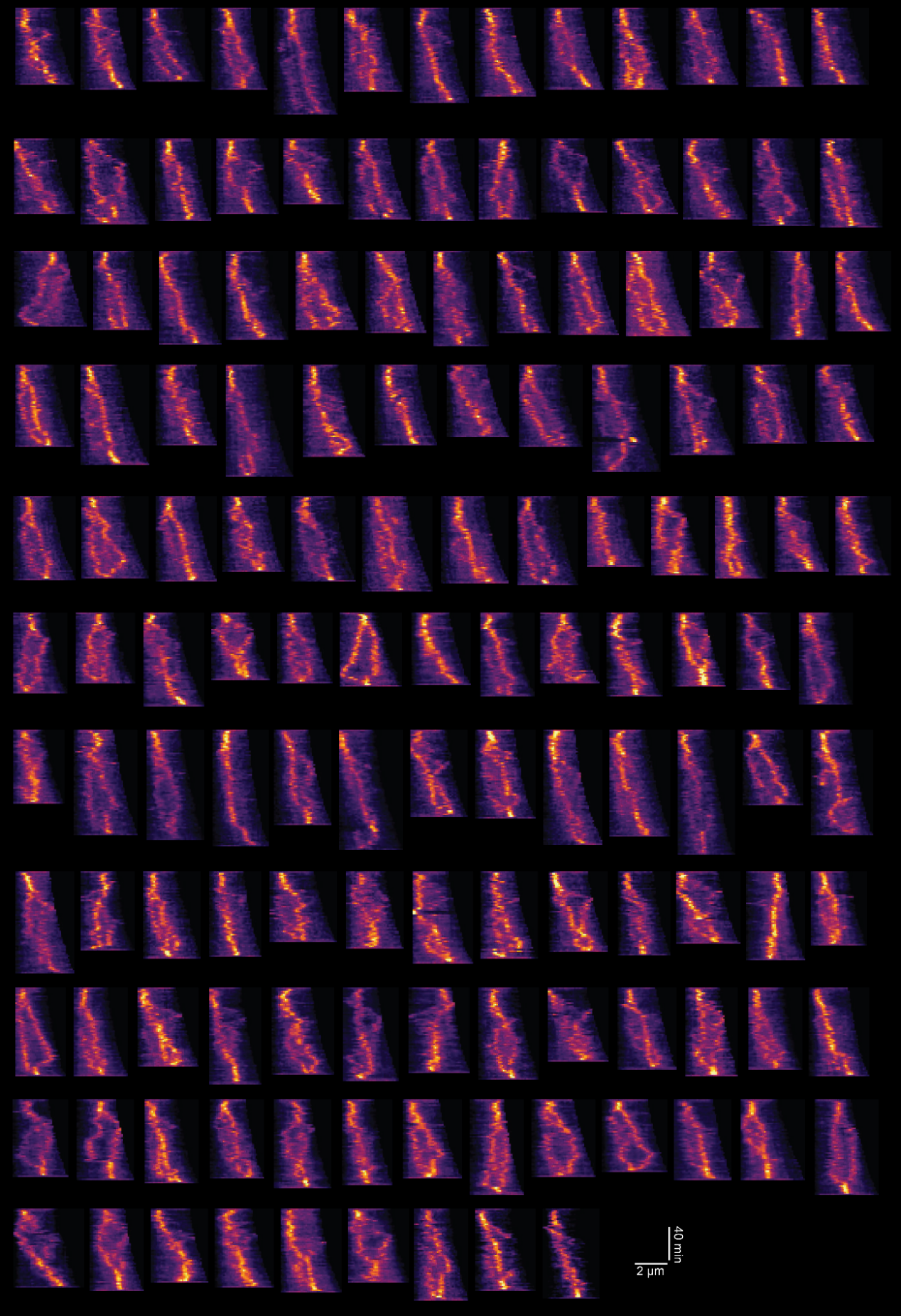


# **Supplementary Figure 20 |** Kymographs of time-lapse imaging of the CB15N *flip1-5::dnaN-sfGFP* cells.


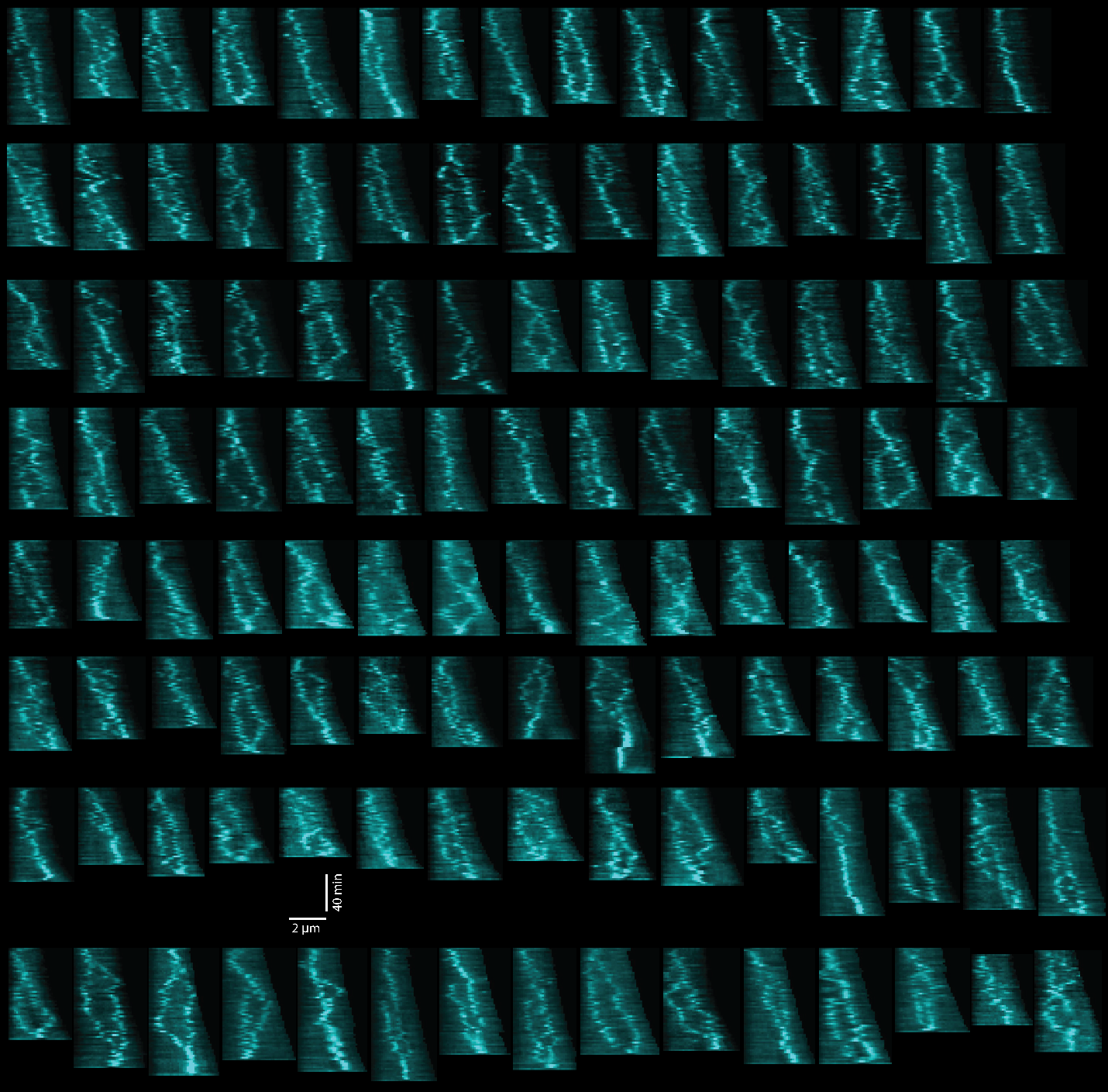


# **Supplementary Figure 21 |** Kymographs of time-lapse imaging of the CB15N *flip1-5::P_xyl_-SSB-sfGFP* cells.


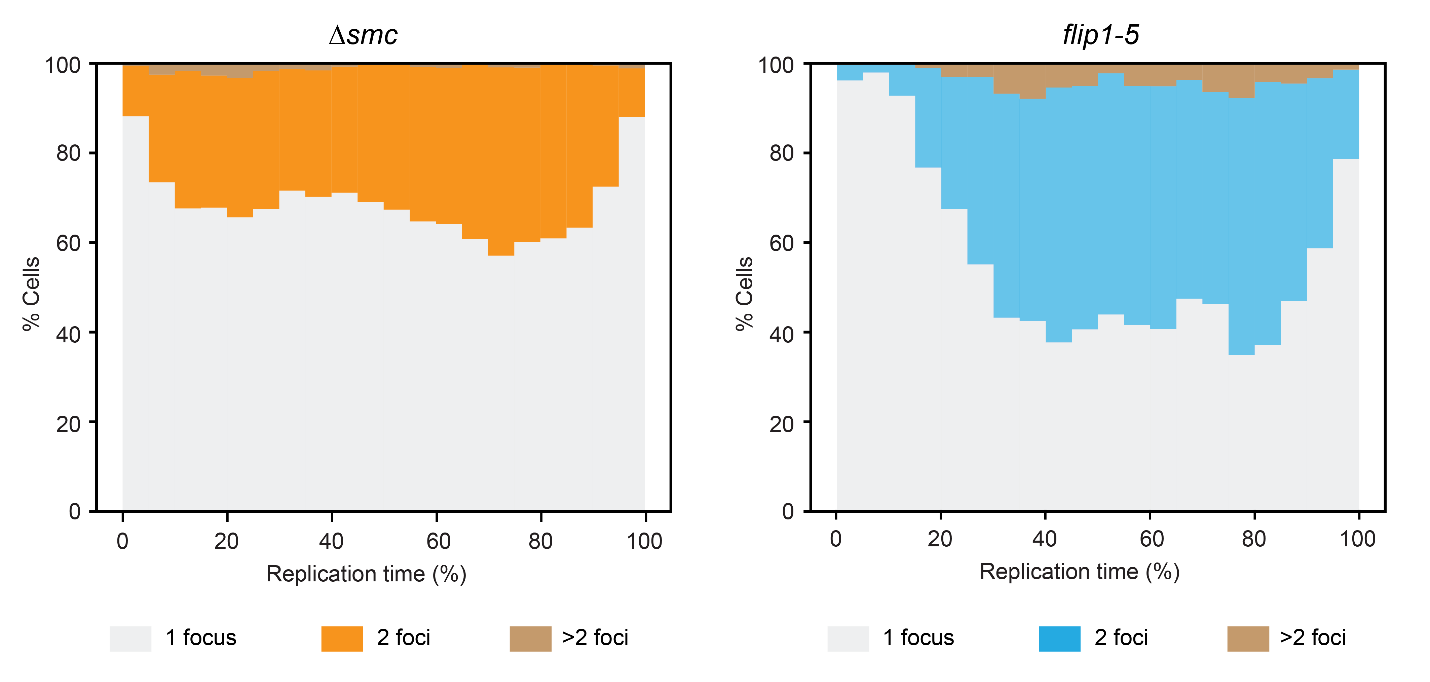


# **Supplementary Figure 22 |** Distribution of CB15N *∆smc::dnaN-sfGFP* (left) and CB15N *flip1-5::dnaN-sfGFP* (right) cells that contain 1, 2, and >2 detected DnaN foci. Source data are provided as a Source Data file.


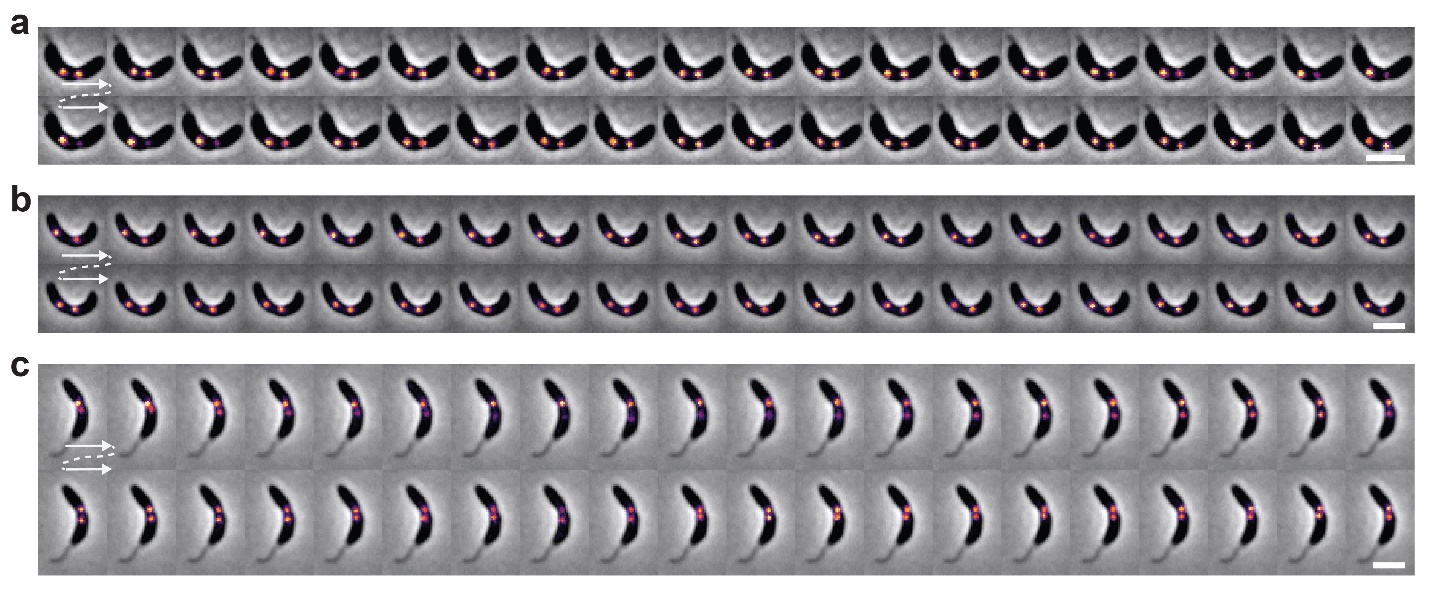


# **Supplementary Figure 23 | a-c** Three time-lapse montages of DnaN-sfGFP fluorescence and phase contrast cell shapes in representative pre-divisional CB15N*::dnaN-sfGFP* cells. Scale bar: 2 µm.

**
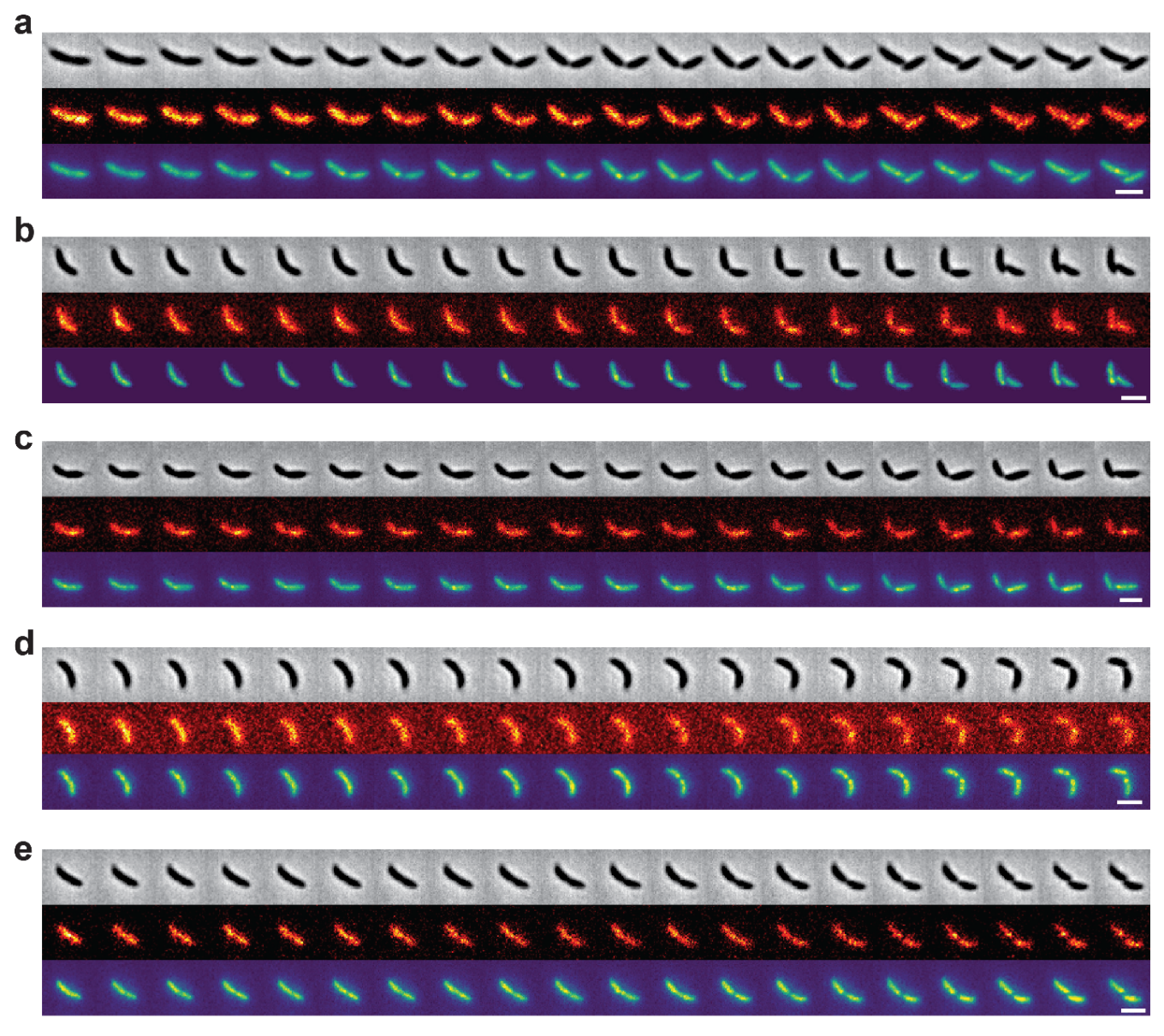
**

# **Supplementary Figure 24 |** **a-e** Five time-lapse montages of phase contrast (top), mCherry-parB^P1^ targeted to L5’ fluorescence image (middle, pseudo-colored in Red-Hot), yGFP-parB^pMT1^ targeted to R5’ fluorescence image (bottom, pseudo-colored in MPI-viridis) of CB15N*::L5’::R5’* cells with 3 min intervals. Scale bar: 2 µm.


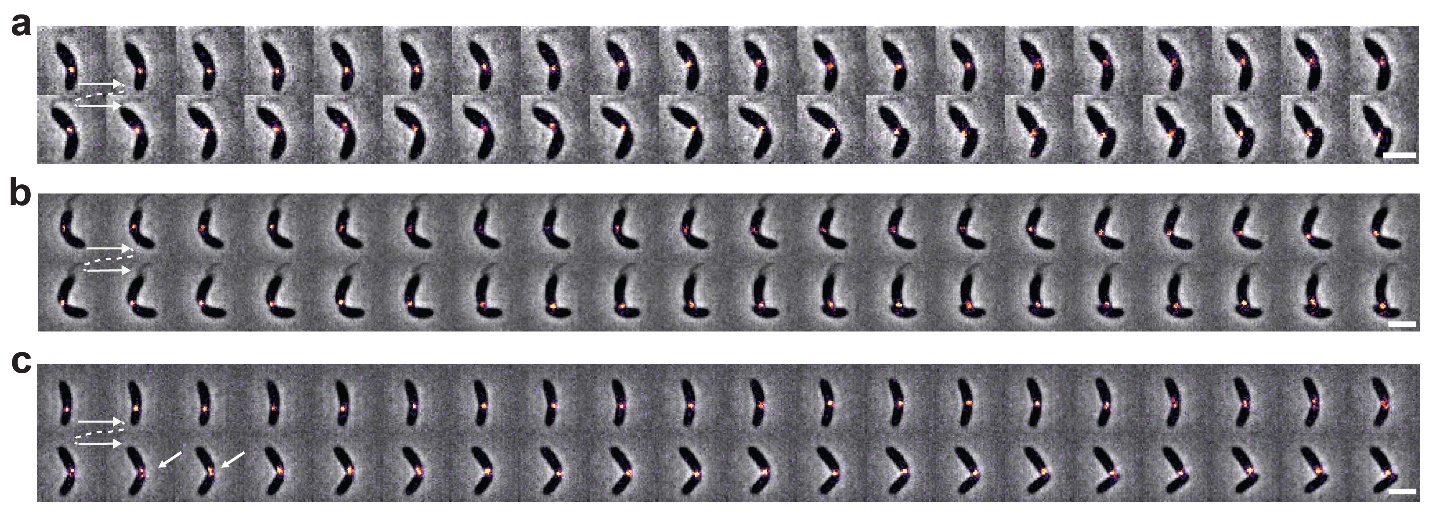


# **Supplementary Figure 25 |** The disappearance of split loci in (**a**) CB15N*::L5* and (**b**-**c**) CB15N*::R5* cells in 2 min interval time-lapse montages. Scale bar: 2 µm.


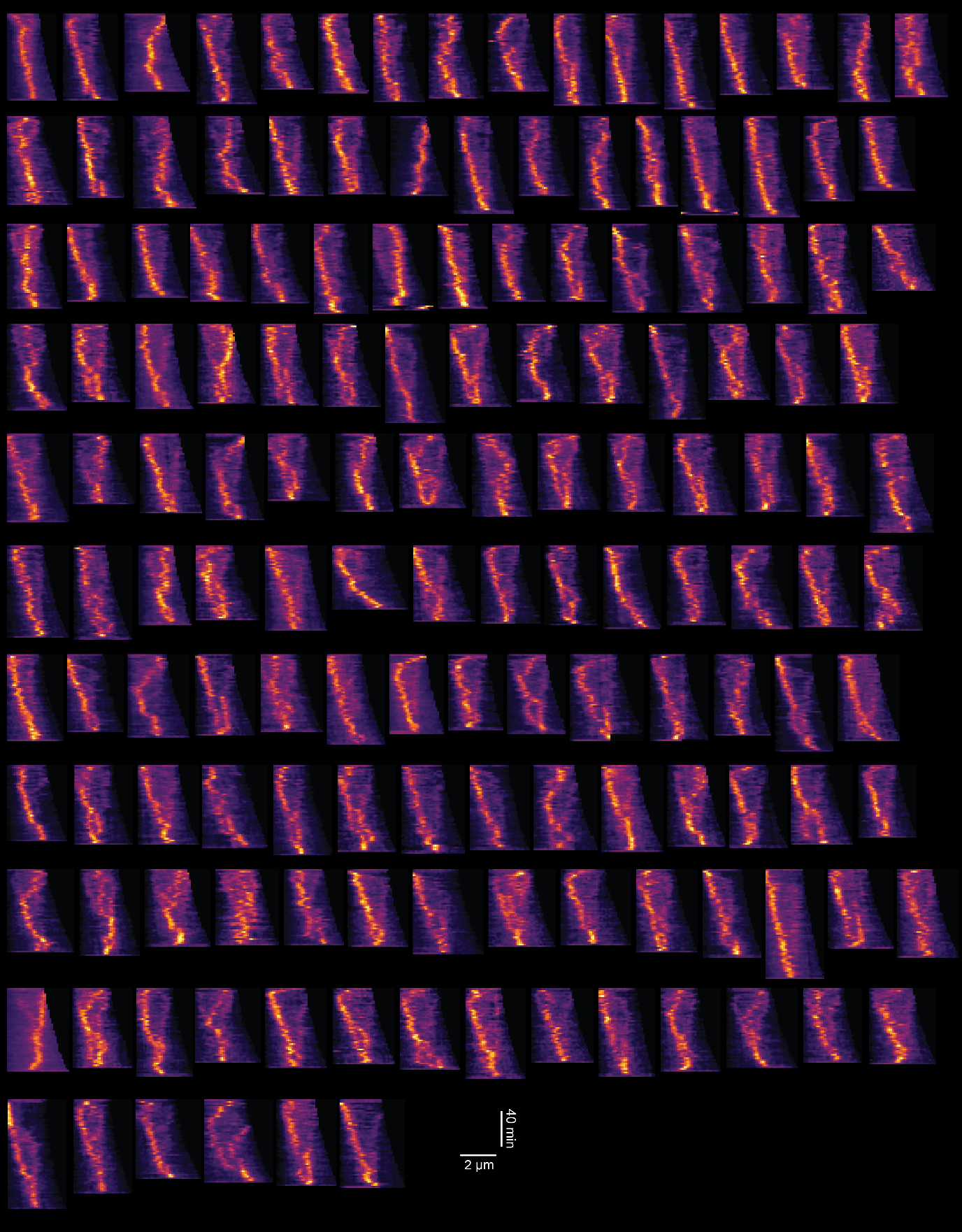


# **Supplementary Figure 26 |** Kymographs of time-lapse imaging of the CB15N *∆rsaA::dnaN-sfGFP* cells.


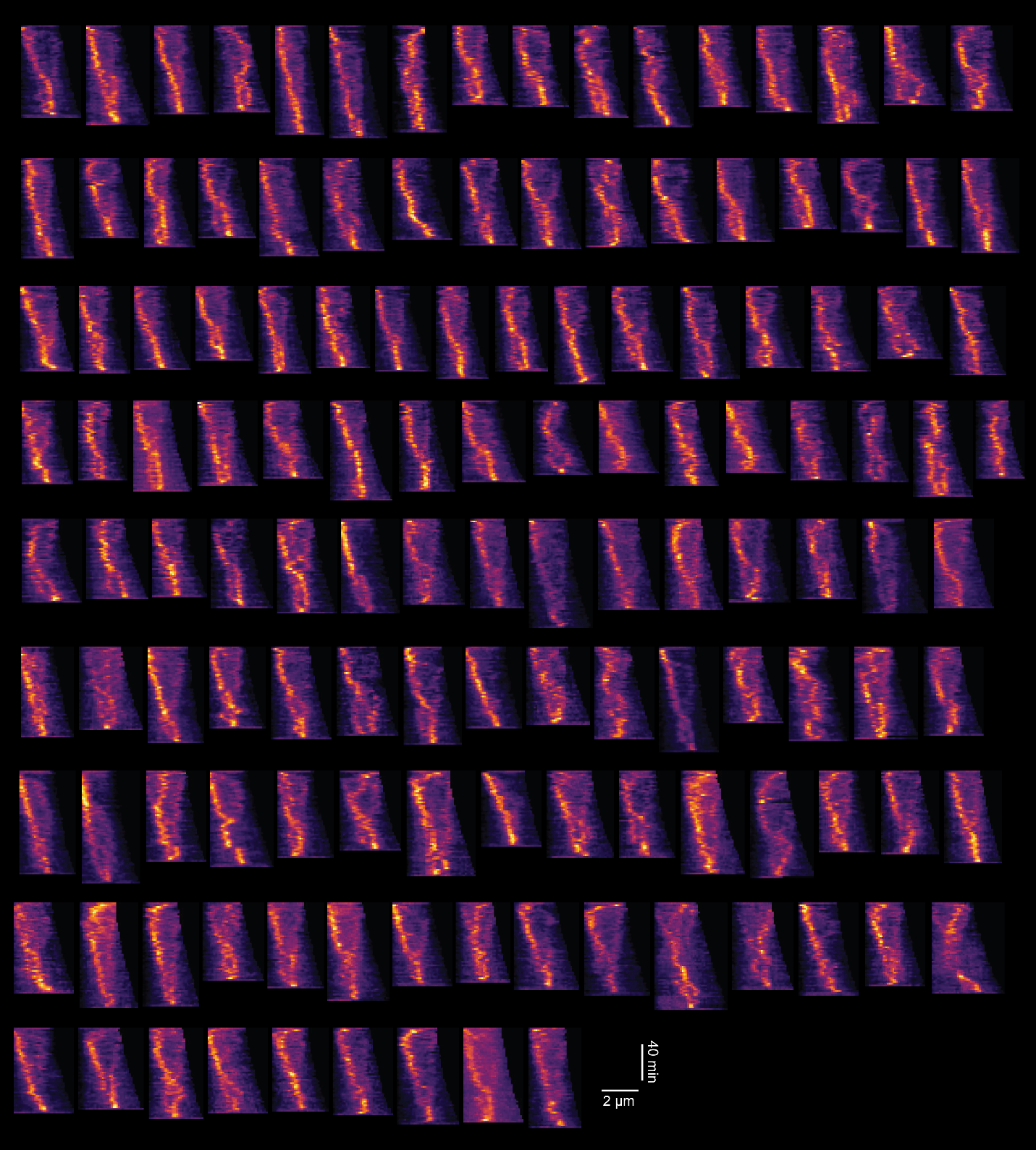


# **Supplementary Figure 27 |** Kymographs of time-lapse imaging of the CB15N *∆rsaA::P_xyl_-rasA::dnaN-sfGFP* cells.


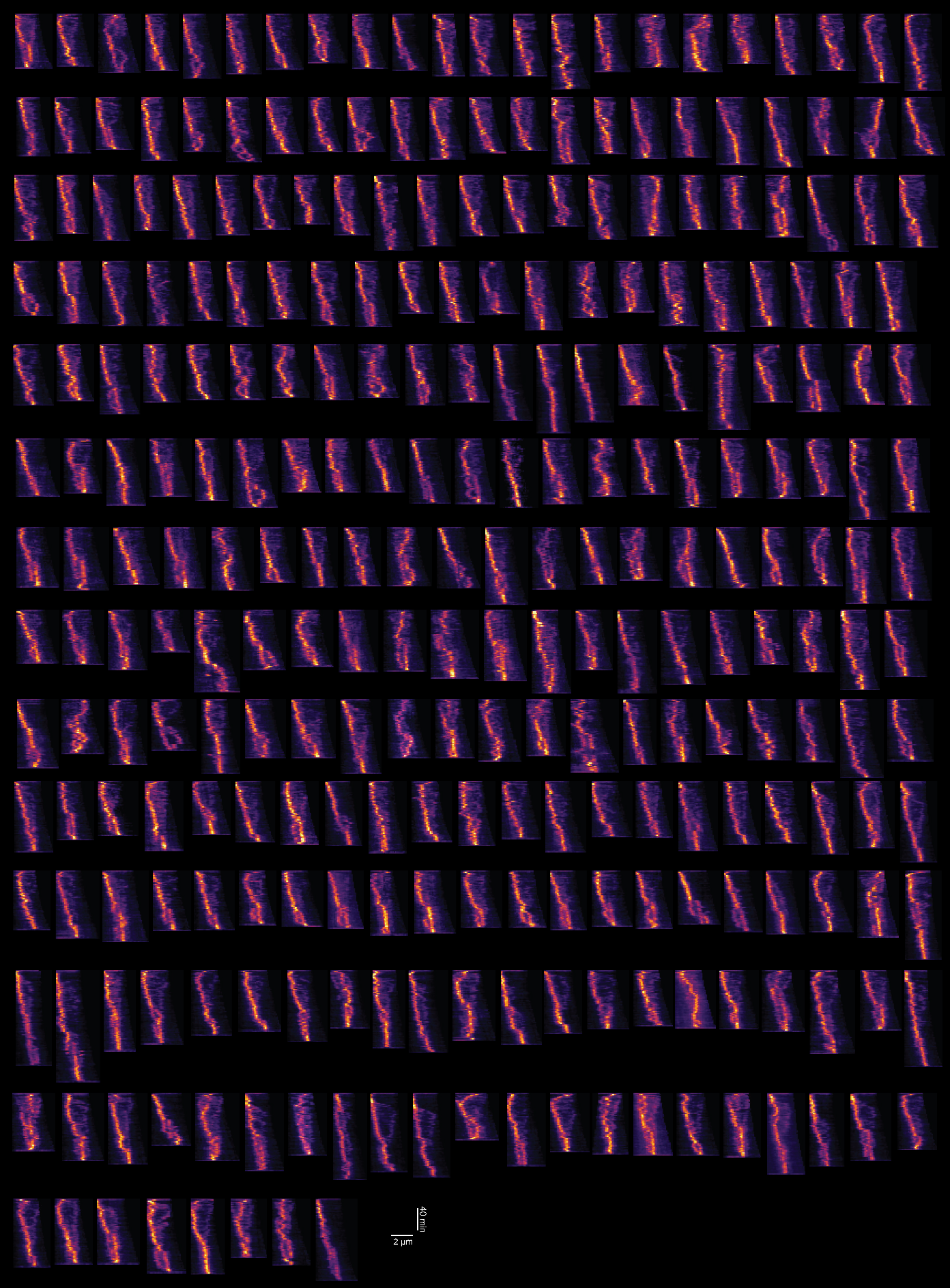


# **Supplementary Figure 28 |** Kymographs of time-lapse imaging of the *rsaA+::dnaN-sfGFP* cells.


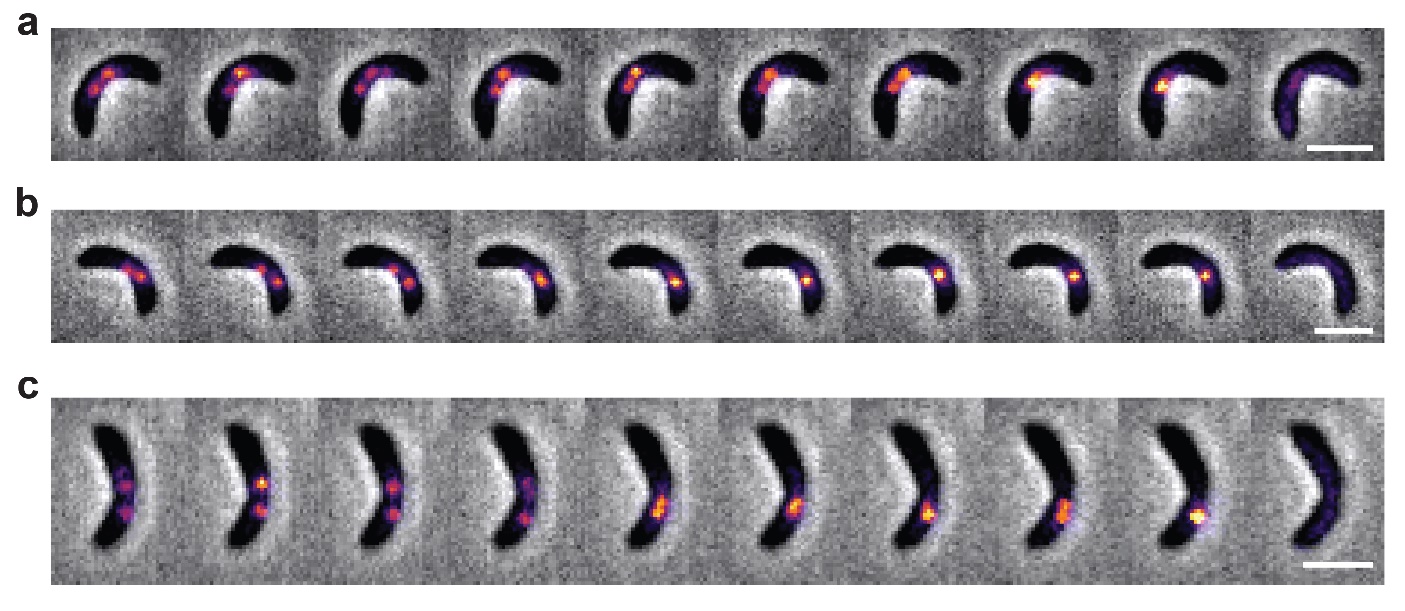


# **Supplementary Figure 29 |** **a-c** Three time-lapse montages of phase contrast and DnaN-sfGFP fluorescence in *rsaA+::dnaN-sfGFP* cells, with a 2 min interval between images. Scale bar: 2 µm.


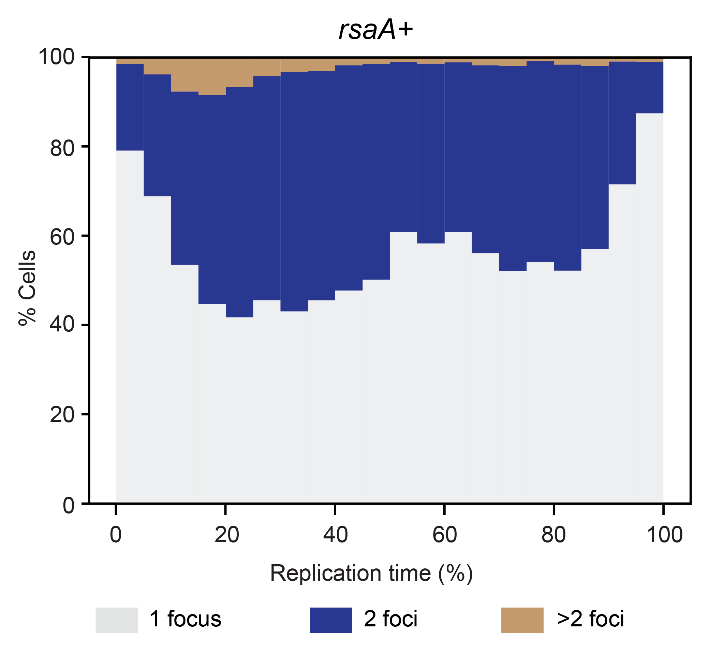


# **Supplementary Figure 30 |** Distribution of *rsaA*+*::dnaN-sfGFP* cells that contain 1, 2, and >2 detected DnaN foci. Source data are provided as a Source Data file.


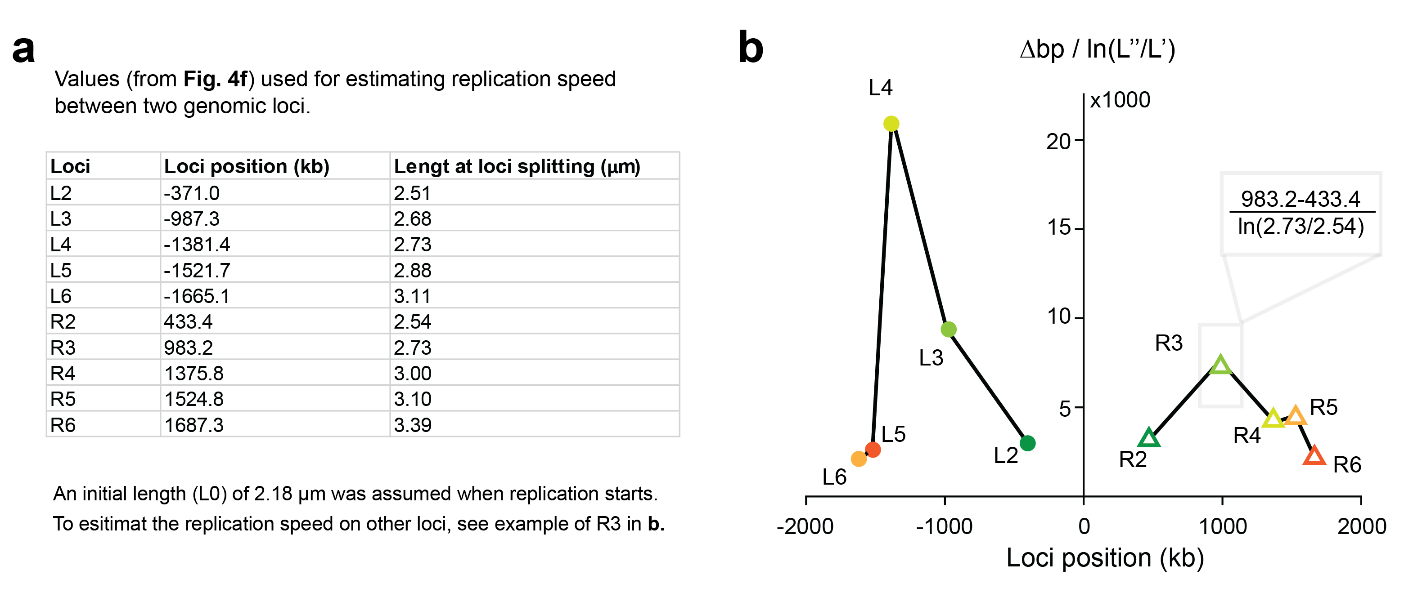


# **Supplementary Figure 31 |** Values of cell length recorded for estimating the replication speed (**a**) and plot of the estimated speed at different genomic loci positions (**b**). Source data are provided as a Source Data file.

**Supplementary References**

1. Collier, J. & Shapiro, L. Feedback Control of DnaA-Mediated Replication Initiation by Replisome-Associated HdaA Protein in Caulobacter. *J. Bacteriol.* **191**, 5706–5716 (2009).

2. Mahecic, D. *et al.* Event-driven acquisition for content-enriched microscopy. *Nat. Methods* **19**, 1262–1267 (2022).

3. Le, T. B. K., Imakaev, M. V., Mirny, L. A. & Laub, M. T. High-Resolution Mapping of the Spatial Organization of a Bacterial Chromosome. *Science* **342**, 731–734 (2013).

4. Thanbichler, M., Iniesta, A. A. & Shapiro, L. A comprehensive set of plasmids for vanillate- and xylose-inducible gene expression in Caulobacter crescentus. *Nucleic Acids Res.* **35**, e137 (2007).

5. Christen, B. *et al.* The essential genome of a bacterium. *Mol. Syst. Biol.* **7**, 528 (2011).
