# Supplementary material for: Chromosome organization shapes replisome dynamics in Caulobacter crescentus
Source: Nat Commun. 2024 Apr 24;15:3460. doi: 10.1038/s41467-024-47849-6 (PMC11043382; doi:10.1038/s41467-024-47849-6)
Supplement: Supplementary file 3 — Description of additional supplementary files [file 41467_2024_47849_MOESM3_ESM.pdf]

Description of Additional Supplementary Files

File Name: Supplementary Data 1

Description: Details of plasmids and strains used in this study

File Name: Supplementary Movie 1

Description: Time-lapse video of *CB15N :: DnaN-sfGFP* cells with a 2 min interval.

File Name: Supplementary Movie 2

Description: Time-lapse video of *CB15N ::* P_{xyl}*-SSB-sfGFP* cells with a 2 min interval.

File Name: Supplementary Movie 3

Description: Time-lapse video of *CB15N* Δ*smc :: DnaN-sfGFP* cells with a 2 min interval.

File Name: Supplementary Movie 4

Description: Time-lapse video of *CB15N flip1-5 :: DnaN-sfGFP* cells with a 2 min interval.

File Name: Supplementary Movie 5

Description: Time-lapse video of *CB15N flip1-5 ::* P_{xyl}*-SSB-sfGFP* cells with a 2 min interval.

File Name: Supplementary Movie 6

Description: Time-lapse video of *CB15N :: DnaN-sfGFP* cells with a 10 s interval.

File Name: Supplementary Movie 7

Description: Time-lapse video of *CB15N* Δ*rsaA :: DnaN-sfGFP* cells with a 2 min interval.

File Name: Supplementary Movie 8

Description: Time-lapse video *CB15N rsaA+:: DnaN-sfGFP* cells with a 2 min interval.
