# Supplementary material for: Chromosome organization shapes replisome dynamics in Caulobacter crescentus
Source: Nat Commun. 2024 Apr 24;15:3460. doi: 10.1038/s41467-024-47849-6 (PMC11043382; doi:10.1038/s41467-024-47849-6)
Supplement: Supplementary file 13 — Reporting Summary [file 41467_2024_47849_MOESM13_ESM.pdf]

nature portfolio

Corresponding author(s): Suliana Manley

Last updated by author(s): Mar 26, 2024

Reporting Summary

Nature Portfolio wishes to improve the reproducibility of the work that we publish. This form provides structure for consistency and transparency in reporting. For further information on Nature Portfolio policies, see our Editorial Policies and the Editorial Policy Checklist.

Statistics

For all statistical analyses, confirm that the following items are present in the figure legend, table legend, main text, or Methods section.

n/a	Confirmed	
☐	☒	The exact sample size (*n*) for each experimental group/condition, given as a discrete number and unit of measurement
☐	☒	A statement on whether measurements were taken from distinct samples or whether the same sample was measured repeatedly
☐	☒	The statistical test(s) used AND whether they are one- or two-sided<br>*Only common tests should be described solely by name; describe more complex techniques in the Methods section.*
☒	☐	A description of all covariates tested
☒	☐	A description of any assumptions or corrections, such as tests of normality and adjustment for multiple comparisons
☐	☒	A full description of the statistical parameters including central tendency (e.g. means) or other basic estimates (e.g. regression coefficient) AND variation (e.g. standard deviation) or associated estimates of uncertainty (e.g. confidence intervals)
☐	☒	For null hypothesis testing, the test statistic (e.g. *F*, *t*, *r*) with confidence intervals, effect sizes, degrees of freedom and *P* value noted<br>*Give P values as exact values whenever suitable.*
☒	☐	For Bayesian analysis, information on the choice of priors and Markov chain Monte Carlo settings
☒	☐	For hierarchical and complex designs, identification of the appropriate level for tests and full reporting of outcomes
☒	☐	Estimates of effect sizes (e.g. Cohen's *d*, Pearson's *r*), indicating how they were calculated

Our web collection on statistics for biologists contains articles on many of the points above.

Software and code

Policy information about availability of computer code

Data collection	Imaging data were collected through VisView 5.0.0.21.
Data analysis	Raw single-cell images were processed by MicrobeJ 5.13n(13) for cell segmentation and foci detection. The template for running MicrobeJ analysis and the data post-processing code (Python 3.7.3) are publicly available from Zenodo: https://doi.org/10.5281/zenodo.10875454.

For manuscripts utilizing custom algorithms or software that are central to the research but not yet described in published literature, software must be made available to editors and reviewers. We strongly encourage code deposition in a community repository (e.g. GitHub). See the Nature Portfolio guidelines for submitting code & software for further information.

Data

Policy information about availability of data

All manuscripts must include a data availability statement. This statement should provide the following information, where applicable:

- Accession codes, unique identifiers, or web links for publicly available datasets
- A description of any restrictions on data availability
- For clinical datasets or third party data, please ensure that the statement adheres to our policy

All raw microscopy data used in the study are publicly available on the Zenodo repository: https://doi.org/l0.5281/zenodo.10203421. The source data underlying Fig. 1e,f; Fig. 3g; Fig. 4e-g; Fig. 5f; Fig. 6e-g are provided as a Source Data file.

Research involving human participants, their data, or biological material

Policy information about studies with human participants or human data. See also policy information about sex, gender (identity/presentation), and sexual orientation and race, ethnicity and racism.

Reporting on sex and gender	Not applicable.
Reporting on race, ethnicity, or other socially relevant groupings	Not applicable.
Population characteristics	Not applicable.
Recruitment	Not applicable.
Ethics oversight	Not applicable.

Note that full information on the approval of the study protocol must also be provided in the manuscript.

Field-specific reporting

Please select the one below that is the best fit for your research. If you are not sure, read the appropriate sections before making your selection.

☒ Life sciences ☐ Behavioural & social sciences ☐ Ecological, evolutionary & environmental sciences

For a reference copy of the document with all sections, see nature.com/documents/nr-reporting-summary-flat.pdf

Life sciences study design

All studies must disclose on these points even when the disclosure is negative.

Sample size	Typically several thousands of cells were selected for the analysis in each experimental condition. The number was chosen at random, depending on the density of the number of cells per image position Typically up to few thousands of cells are reported in similar replisome studies.
Data exclusions	Imaging data were excluded if the detected DnaN foci failed with a Gaussian fitting, for instance, zero amplitude, extreme small (<l) or large (>3) sigma values, or low signal-to-noise ratio (amplitude/offset< 0.25).
Replication	Each experiment was reproduced with three biological repeats. All replications were successful.
Randomization	Samples were allocated to experimental groups based on the growth conditions (growth temperature, growth media, presence of antibiotics etc.) used. No randomization was not relevant.
Blinding	Blinding was not performed, because depending on the growth conditions (growth temperature, growth media, presence of antibiotics) the cell morphology and physiology were clearly different between cells, making the blinding not possible.

Reporting for specific materials, systems and methods

We require information from authors about some types of materials, experimental systems and methods used in many studies. Here, indicate whether each material, system or method listed is relevant to your study. If you are not sure if a list item applies to your research, read the appropriate section before selecting a response.

Materials & experimental systems

n/a	Involved in the study
☒	☐ Antibodies
☒	☐ Eukaryotic cell lines
☒	☐ Palaeontology and archaeology
☒	☐ Animals and other organisms
☒	☐ Clinical data
☒	☐ Dual use research of concern
☒	☐ Plants

Methods

n/a	Involved in the study
☒	☐ ChIP-seq
☒	☐ Flow cytometry
☒	☐ MRI-based neuroimaging

Plants

Seed stocks	Not applicable.
Novel plant genotypes	Not applicable.
Authentication	Not applicable.
